# Chemosensitivity of U251 Cells to the Co-treatment of D-Penicillamine and Copper: Possible Implications on Wilson Disease Patients

**DOI:** 10.3389/fnmol.2017.00010

**Published:** 2017-01-31

**Authors:** Meghri Katerji, Kassem Barada, Mustapha Jomaa, Firas Kobeissy, Ahmad-Kareem Makkawi, Wassim Abou-Kheir, Julnar Usta

**Affiliations:** ^1^Department of Biochemistry and Molecular Genetics, Faculty of Medicine, American University of BeirutBeirut, Lebanon; ^2^Department of Internal Medicine, American University of Beirut Medical CenterBeirut, Lebanon; ^3^Department of Anatomy, Cell Biology and Physiological Sciences, Faculty of Medicine, American University of BeirutBeirut, Lebanon

**Keywords:** copper, Wilson disease, ceruloplasmin, D-penicillamine, U251, PC12, SH-SY5Y

## Abstract

D-Penicillamine (PA), a copper chelator, and one of the recommended drugs for treatment of Wilson disease (WD) has been reported to worsen the symptoms of patients with neurologic presentations. However, the cause of this paradoxical response has not been fully elucidated and requires further investigations. Accordingly, we have studied the *in vitro* effect of Copper (Cu) and/or PA treatment on human glioblastoma U251 cells as an *in vitro* model of Cu cytotoxicity. Treatment of U251 cells with either Cu or PA exerted no significant effect on their morphology, viability or ROS level. In contrast, co-treatment with Cu-PA caused a decrease in viability, altered glutathione and ceruloplasmin expression coupled with marked increase in ROS; depolarization of mitochondrial membrane potential; and an increase in Sub G0 phase; along with alpha-Fodrin proteolysis. These findings along with the absence of LDH release in these assays, suggest that combined Cu-PA exposure induced apoptosis in U251 cells. In addition, pre-/or co-treatment with antioxidants showed a protective effect, with catalase being more effective than N-acetyl cysteine or trolox in restoring viability and reducing generated ROS levels. By comparison, a similar analysis using other cell lines showed that rat PC12 cells were resistant to Cu and/or PA treatment, while the neuroblastoma cell line SH-SY5Y was sensitive to either compound alone, resulting in decreased viability and increased ROS level. Taken together, this study shows that glioblastoma U251 cells provide a model for Cu-PA cytotoxicity mediated by H_2_O_2_. We postulate that PA oxidation in presence of Cu yields H_2_O_2_ which in turn permeates the plasma membrane and induced apoptosis. However, other cell lines exhibited different responses to these treatments, potentially providing a model for cell type- specific cytotoxic responses in the nervous system. The sensitivity of different neural and glial cell types to Cu-PA treatment may therefore underlie the neurologic worsening occurring in some PA-treated WD patients. Our results also raise the possibility that the side effects of PA treatment might be reduced or prevented by administering antioxidants.

## Introduction

Copper (Cu) is a ubiquitous trace element stored primarily in the liver, but also present in other organs, such as the brain, heart, kidney, and muscles (Osredkar and Sustar, 2011). It is an essential micronutrient required for the catalytic and structural properties of several important enzymes including: cytochrome c oxidase (Yoshikawa et al., 1995; Tsukihara et al., 1996); ceruloplasmin (Cp) (Kaplan and O'Halloran, 1996); dopamine-β-monooxygenase (Rahman et al., 2009); Cu-Zn dependent superoxide dismutase (Tainer et al., 1983); and peptidylglycine-α-monooxygenase (Bousquet-Moore et al., 2010). However, having unpaired electrons, excess copper generates highly toxic hydroxyl and superoxide free radicals favoring lipid peroxidation, mitochondrial impairment, DNA strand breakage, and protein damage (Halliwell and Gutteridge, 1990; Fraga, 2005). Thus, the *in vivo* regulation of copper levels in biological systems is under strict control through the actions of copper transporters and chaperones (Harris, 2000; Madsen and Gitlin, 2007; Robinson and Winge, 2010; Jiang et al., 2013).

Defects in the ATP7B gene encoding a copper transporting Cu-ATPase disrupt the homeostatic copper balance leading to Wilson disease (WD), that is characterized by reduced biliary Cu excretion, and impaired Cu incorporation into Cp (Cox and Moore, 2002; de Bie et al., 2007; Lutsenko et al., 2007). Loading of copper into apo-Cp occurs in the trans-Golgi network yielding the active holo-Cp, the main plasma copper transporting protein in circulation (Terada et al., 1998; Meyer et al., 2001). Hence, failure of Cp-metallation and biliary copper excretion results in copper accumulation primarily in the liver and brain leading to hepatic cirrhosis and/or progressive basal ganglia degeneration in WD patients (Madsen and Gitlin, 2007). The therapeutic objective in the treatment of WD patients is to restore normal copper homeostasis by either reducing the absorption of dietary copper, or promoting its excretion (Gilroy et al., 2016).

**Figure 1:**
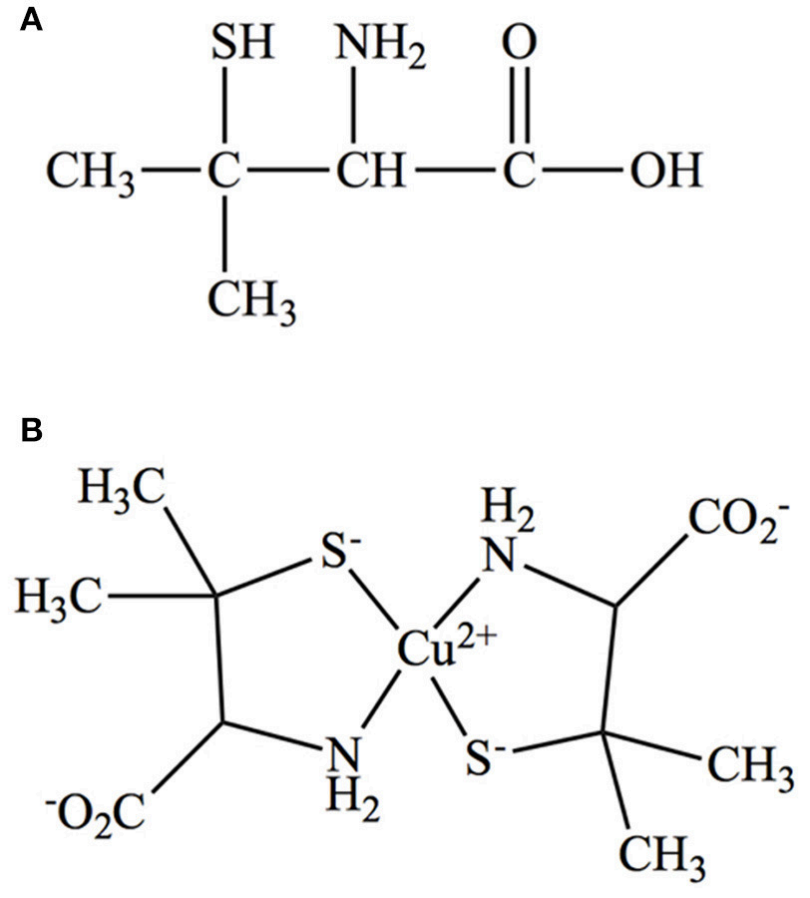
**(A)** Structure of D-Penicillamine. **(B)** Structure of Cu-PA ring complex.

D-Penicillamine (PA) (Figure 1A), first identified as a product of penicillin hydrolysis, is the drug of choice to treat WD patients, is marketed as Cuprimine or Depen (Stephenson and Roberson, 1960). Following its absorption through the gastrointestinal tract (Van Caillie-Bertrand et al., 1985), PA binds excess copper via its sulphydryl (SH) and amino (NH_2_) groups forming a non-toxic ring complex (Figure 1B; Walshe, 2009). Furthermore, it mobilizes intracellular copper into circulation for later excretion in urine (McArdle et al., 1990). However, like any other drug, PA has a number of side effects ranging from loss of taste, headache, and abdominal pain to more serious problems including hypersensitivity, suppression of bone marrow, skin toxicity, nephro-toxicity, and autoimmune diseases (Scheinberg et al., 1987; Czlonkowska et al., 1997).

More importantly, during the early stage of administration, PA has been reported to result in severe deterioration in about 50% of WD patients with neurologic symptoms with minimal recovery even following drug discontinuation (Brewer et al., 1987; Kalita et al., 2014). Being a pyridoxine (Vitamin B6) antagonist, PA leads to the depletion of Vitamin B6, forming a thiazolidine derivative (Walshe, 2011). Other studies performed on toxic milk mice, WD animal model, reported that PA mobilization of serum and brain copper decrease the protein-bound copper concentration and increase the oxidative stress in the brain (Chen et al., 2012). Free and loosely bound copper contributes to free radical production (Ogihara et al., 1995) that perturbs antioxidants' status and induces neurodegenerative disorders in humans (Gilgun-Sherki et al., 2001). However, evaluation of the systemic antioxidant potential of WD patients treated with de-coppering agents, such as PA, showed some improvement without restoring the normal capacity of antioxidant parameters (Gromadzka et al., 2014). The exact mechanisms underlying the worsening of the neurological symptoms in PA-treated WD patients remain unclear and not fully elucidated yet, requiring further investigations. Hence, our study aims at assessing the effect of this copper chelating agent on neural cell lines *in vitro*.

We hereby report the effect of Cu-PA co-treatment on glioblastoma (U251), and neuroblastoma (SH-SY5Y) and pheochromocytoma (PC12) cells. U251 cells were the most sensitive to Cu-PA co-treatment while PC12 were least sensitive. Concomitant to the decrease in viability of U251 cells, we obtained a remarkable increase in ROS, a decrease in GSH levels and increase in SubG0 (99%). While pre-treatment with catalase reversed completely the marked apoptotic phenotype, N-acetyl cysteine and Trolox ameliorated partially the Cu-PA effect suggesting a key role for hydrogen peroxide (H_2_O_2_) in the induced apoptosis. The Cu-PA effect was not specific to human glioblastoma cell line only but was also shown in SH-SY5Y, a neuroblastoma cell line. However, our preliminary results with the latter cell line are suggestive of a different mechanism than that seen in U251 cells. Findings of the current study have identified potential targets for Cu-PA cytotoxicity and shed some light on one of the underlying possible mechanisms that worsen the neurologic manifestations of D-PA treated WD patients. In addition the demonstrated protective effects of antioxidants (viability and ROS) pose the question as to whether antioxidant supplements be part of therapeutic regimen when PA -treated WD patients.

## Materials and methods

### Reagents and antibodies

The following reagents were purchased from the indicated suppliers: *Sigma-Aldrich (Missouri, USA):* Dulbecco's Modified Eagle's medium (DMEM, and DMEM F12-HAM), Heat Inactivated Horse Serum (HS) Donor Herd, Heat Inactivated Fetal Bovine Serum (FBS), Ribonuclease A (RNase A) (cat# R6513), Propidium Iodide (PI) (cat# P4170), Nitro-Blue-Tetrazolium (NBT) Chloride, D-PA (cat# P4875), 0.4% Trypan Blue Solution, Penicillin-Streptomycin (PS) mixture, Catalase, NAC; *Acros Organics (New Jersey, USA)*: Trolox 97%; *Lonza (Maryland, USA)*: Phosphate buffered saline (PBS) without calcium and magnesium, 10X Trypsin; *Biorad (California, USA)*: Biorad protein assay kit, 4X Laemmli protein sample buffer, Enhanced Chemi Luminescence's Reagent (ECL) kit (cat# 1705060); *Roche (Germany):* Cytotoxicity Detection Kit^PLUS^ for lactate dehydrogenase (LDH) release (cat# 04744934001), Cell Proliferation Kit I for 3-(4,5-dimethylthiazol-2-yl)-2,5-diphenyltetrazolium bromide (MTT) assay (cat#11465007001); *Immunochemistry Technologies LLC (Minnesota, USA):* MitoPT™ Kit for 5, 5′, 6, 6′–tetrachloro–1, 1′, 3, 3′–tetraethyl–benzamidazolocarbocyanin iodide (JC-1) assay (cat# 911); *GE Healthcare (UK)*: Rainbow molecular ladder (RPN800E); *Abcam (UK):* Fluorometric Glutathione (GSH) Detection Assay Kit (ab65322), rabbit anti-ceruloplamin antibody (ab131220); *Enzo Life Sciences (New York, USA):* mouse anti-α-Fodrin antibody (BML-FG6090); *Santa Cruz (California, USA):* mouse anti-GAPDH antibody (Sc-32233); *Jackson ImmunoResearch (Pennsylvania, USA)*:Peroxidase-conjugated goat anti-mouse IgG (JIR-115-035-166), Peroxidase-conjugated goat anti-rabbit IgG (JIR-111-035-003).

### U251 cell culture and treatment

U251 (formerly U373) human glioblastoma astrocytoma cells (passages ranging from 20 to 45), provided as a gift by Dr. Firas Kobeissy, were cultured in DMEM media supplemented with 10% FBS, and 0.5% PS. Cells were seeded in 96-well plates (0.8 × 10^4^ cells/well/100 μl media), 12-well plates (0.8 × 10^5^ cells/well/1 mL media), or 100 mm petri-dishes (0.8 x 10^6^ cells/10 mL media), incubated in a humidified 5% CO_2_ incubator at 37°C, and then treated for 24 h with varying CuSO_4_ concentrations to determine IC_50_. In addition, cells were treated with PA (250 μM) or a premix of Cu-PA (IC50, 250 μM) to investigate the protective effect of PA as a copper chelator.

### MTT cell proliferation assay

The viability of treated U251 cells was initially assessed in 96-well plates using the colorimetric MTT cell proliferation assay kit, following the supplier instructions. Briefly, 10 μl of the yellow tetrazolium salt was added to each well of control or treated cells and incubated for 4 h at 37°C. The formazan crystals formed by the mitochondrial dehydrogenase of the metabolically active cells were solubilized overnight by adding 100 μl of solubilizing reagent. The absorbance of the developed color was measured using Multiskan EX ELISA reader at 595 nm. Cell viability was expressed as percentage viability compared to a control of cells treated with equal volume of vehicle (H_2_O).

### Trypan blue exclusion test

To confirm MTT results, the viability of Cu and/or PA treated cells seeded in 100 mm petri-dishes was analyzed using trypan blue exclusion test. While viable cells remain unstained, those with damaged membrane stain blue. Treated cells, collected following trypsinization, were centrifuged (swinging bucket centrifuge) at 900 rpm for 5 min, re-suspended in 1 ml of media, mixed with at a ratio of v/v with 0.4% trypan blue solution, and then were counted under a light microscope using a hemocytometer. The ratio of unstained cells to the total number of cells (stained and unstained) was used to determine the percentage of cell viability.

### NBT reduction assay

Levels of intracellular reactive oxygen species (ROS) in Cu and/or PA treated cells seeded in 96-well plates were determined using NBT assay (Muñoz et al., 2000). Media of control and treated cells was initially aspirated, followed by the addition of NBT (100 μl of 1 mg/ml) and incubation (1 h, 37°C) in a humidified 5% CO_2_ incubator. The wells were washed with methanol (100 μl), allowed to dry at room temperature, and NBT salts reduced into formazan crystals were then solubilized by the successive addition of KOH (120 μl, 2 M) and DMSO (140 μl). The intensity of the developed blue-turquoise color was quantified using Multiskan EX ELISA reader at 630 nm and the percentage of reduced NBT was calculated from the ratio of absorbance of treated to untreated control cells. The level of ROS generated is inversely proportional to the level of NBT reduced.

### LDH release assay

The extent of necrosis in cells treated with PA and/or Cu was examined in 96-well plates using the Cytotoxicity Detection kit^PLUS^ following the manufacturer's instructions. Rate of cell lysis was determined by colorimetric measure (at 490 nm using Multiskan ELISA) of the amount of LDH released from the cells into the culture medium. The percentage LDH release was quantified as described by the cytotoxicity kit manual using suggested proper controls.

### Fluorescent MitoPT-JC1 assay

The effect of Cu and/or PA on the mitochondrial membrane potential was examined using the cell permeable lipophilic fluorescent JC-1 dye of MitoPT-JC1 assay. Entry of the positively charged JC-1 is favored by the negatively charged non-apoptotic mitochondria yielding red-orange fluorescent aggregates; whereas in compromised cells, JC-1 gets dispersed throughout the cell forming green fluorescent monomers. Treated U251 cells, seeded in 12-well plates on sterile glass cover slips, were stained for 15 min at 37°C with 300 μl of 1X MitoPT-JC1 stain solution, washed twice, and then visualized using a fluorescent microscope (OLYMPUS; BH2-RFCA) containing long band path emission filters Ex 490 nm and Em > 510 nm. Images were taken by OLYMPUS DP71 camera using the DP controller (OLYMPUS, 2001-2006; 3.1.1.267) acquisition software.

### Cell cycle analysis

The effect of the treatments on cell cycle progression was assessed by flow cytometry. The fluorescence intensity of PI-stained DNA reflects the DNA content of a cell, which determines the proportion of cells in the different cell cycle phases. U251 cells were seeded in 100 mm petri-dishes, treated with Cu, PA, or Cu-PA for 24 h, trypsinized, centrifuged (swinging bucket centrifuge), washed with PBS, fixed in 70% ethanol (1 h, −20°C), then allowed to warm room temperature, washed with PBS, re-suspended in RNase A (100 μl of 200 μg/ml), and incubated (1 h, 37°C). Cells were later centrifuged (mini spin centrifuge), re-suspended in PBS (350 μl), and stained by PI (20 μl of 1 mg/mL) for 10 min in the dark, as described by Wlodkowic et al. (2009). The PI-elicited fluorescence was measured using Guava EasyCyte8 Flow Cytometer. Each sample was collected as 5000 ungated events and the corresponding cell cycle distribution, according to the DNA content, was then determined.

### Glutathione level determination

The total intracellular GSH level in PA and/or Cu treated cells was assessed using fluorometric glutathione detection assay kit. Treated and untreated U251 cells, seeded in 100 mm petri-dishes, were collected in eppendorf tubes by centrifugation (700 g, 5 min–mini spin centrifuge), lysed (100 μl of the kit's cell lysis buffer), incubated on ice (10 min), and centrifuged at top speed (13400 rpm, 10 min–mini spin centrifuge). Different volumes of the obtained supernatant, adjusted to a final volume of 100 μl with lysis buffer, were introduced in clear-bottom 96-well plates, followed by the consecutive addition of Glutathione S-transferase (2 μL) and monochlorobimane (2 μL), and incubation for 1 h (37°C). Fluorescence was measured using Fluoroscan Ascent FL at Ex/Em = 360 ± 20 /460 ± 20 nm. GSH levels in Cu, PA, or Cu-PA treated cells were compared to those of untreated control cells.

### Western blot analysis

Western blot analysis was performed to determine the expression of Cp level and to assess the pro-apoptotic marker cleavage of α-Fodrin in Cu, PA, Cu-PA treated cells. Cells were harvested and lysed by Triton 1%-SHT (Sucrose 250 mM–Hepes 10 mM–Tris 50 mM) lysis buffer (pH = 7.4). Protein concentration was quantified (Bio-Rad Bradford assay) using a UV-VIS scanning Spectrophotometer (SHIMADZU–UV–2101 PC). Following standard protocols, lysates containing 75–100 μg of protein were mixed with 4X laemmli buffer, boiled (10 min), and then were separated on 6% and 8% SDS-polyacrylamide gels for α-Fodrin and Cp, respectively. Proteins were then transferred onto a nitrocellulose membrane using BIORAD electro-transfer set up. The membrane was then blocked with 10% fat-free milk for 1 h, and immunoblotted overnight at 4°C with the appropriate primary antibody diluted as follows: anti-Cp (1:300), anti-fodrin (1:500), and anti-GAPDH (1:1000). After three washes (20 min each) with Tris-buffered saline containing 0.1% Tween 20, the membrane was incubated (1 h) with peroxidase-conjugated secondary goat anti-mouse (1:5000) or anti-rabbit (1:5000) and washed again three times. The protein expression was visualized by the ECL reagent using RP X-OMAT processor (Kodak, model M6B), scanned, and quantified using Image J software. Fold expression was determined relative to the control after normalizing for equal loading to their respective GAPDH band.

### Effect of antioxidants on Cu-PA treated U251 cells

The protective effect of antioxidants NAC/Trolox as well as catalase enzyme on viability and ROS generation was investigated using MTT cell proliferation assay and NBT reduction, respectively. Seeding was performed according to the respective assay conducted. U251 cells were co-treated with catalase enzyme (500U) and Cu-PA for 24 h or were pretreated with NAC (5 mM) and Trolox (100 μM) for 2 h, following which, the pretreatment was aspirated and cells were treated with Cu-PA for 24 h. To further assess whether the restoration of viability and oxidative stress by catalase enzyme is dose dependent or independent, varying concentrations of catalase ranging from 1 to 500U were used.

### PC12 cell line

To study the effect of Cu and/or PA on neuronal cells, rat pheochromocytoma cells (PC12) were used (passages ranging from 20 to 45). Cells were cultured with DMEM media supplemented with 10% FBS, 2.5% HS, and 0.5% PS, seeded in 96-well plates (1 × 10^4^ cells/well/100 μl media), 12-well plates (1 × 10^5^ cells/well/1 mL media), or 100 mm petri-dishes (1 × 10^6^ cells/10 mL media) and treated for 24 h with the appropriate concentrations of CuSO_4_ and/or PA. All carried out experiments on U251 cells were repeated on PC12 cell line.

### SH-SY5Y cell lines

The effect of copper and/or PA on viability and reactive oxygen species was also examined using the human neuroblastoma SH-SY5Y cells cultured in DMEM Ham's F-12 (sigma-Aldrich) media supplemented with 10% FBS, and 1% PS. Cells were seeded in: 96 well plates (5 × 10^3^ cells/well/100 ul); and 6 well plates (1 × 10^5^ cells/well/1 ml), treated for 24 h with the appropriate concentrations of CuSO_4_ and/or PA following which NBT reduction assay, and viability (trypan blue) were performed, respectively, as described in previous sections

### Statistical analysis

SPSS software was used to analyze data and determine statistical significance. Both One-Way Anova and Independent sample *t*-test were applied. For One-Way Anova analysis, *Post-hoc* test (Bonferroni and Tukey) were used for multiple comparisons. *P* < 0.05 is considered significant. For each parameter tested, a set of at least three different experiments, unless otherwise mentioned, were done. Moreover, both inter-categorical statistical significance and significance relative to control were analyzed for each parameter. Data are represented as the mean ± standard error of the mean (SEM).

## Results

### *In vitro* cytotoxicity of CuSO_4_ and/or PA

**Figure 2:**
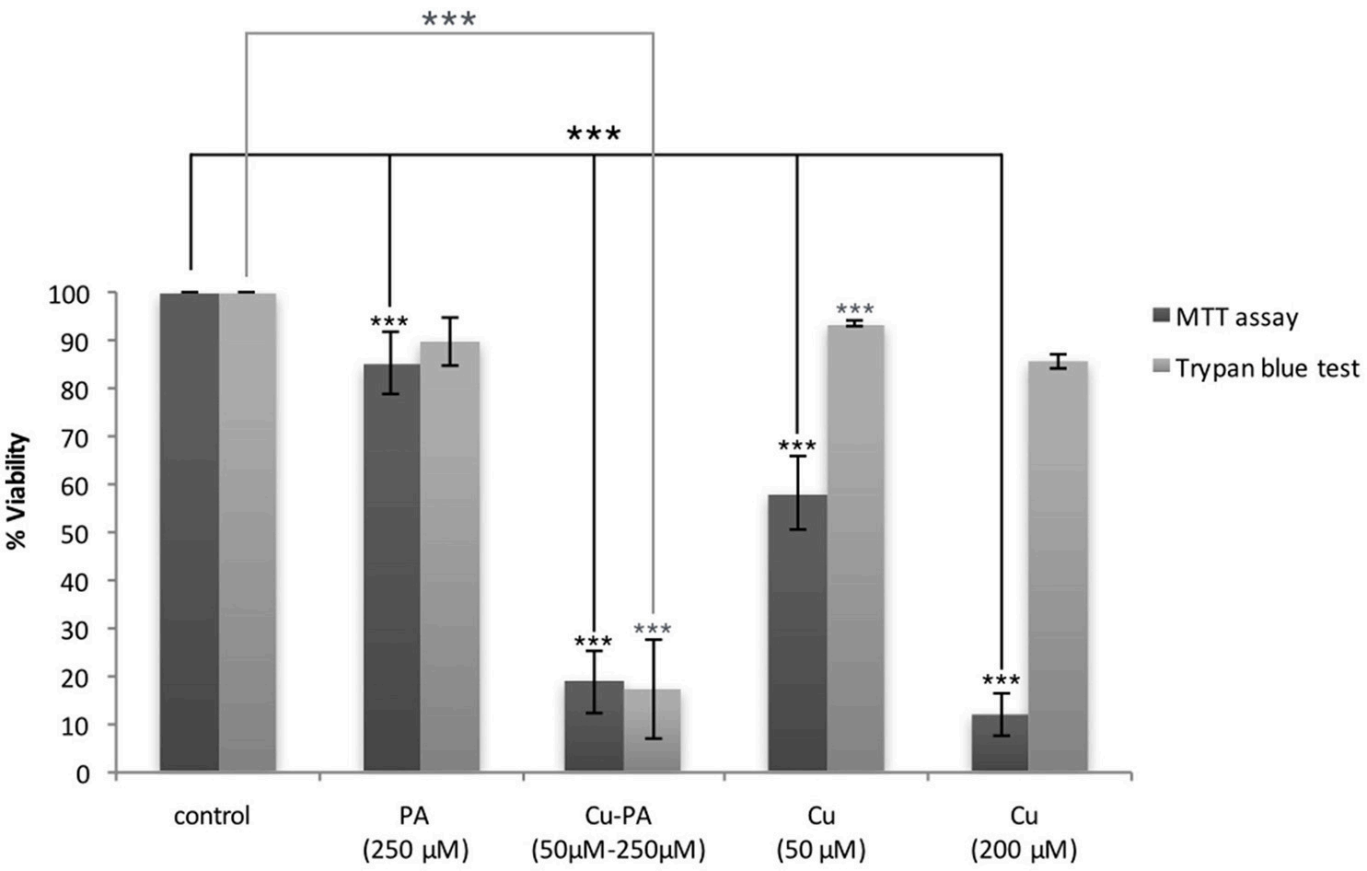
**MTT vs. Trypan blue exclusion assay: Comparative effect of Cu and/or PA on viability of U251 cells**. U251 Cells (0.8 × 10^4^) were treated with CuSO_4_ (50 μM, 200 μM), PA (250 μM), or Cu-PA (50–250 μM) for 24 h. Data presented as % viability is the mean ± SEM of 18-determinations from 6-different experiments using MTT assay, and of 3-determinations from 3- different experiments using Trypan blue exclusion assay. Asterisks on bars represent inter-categorical statistical significance (each category with the preceding one), and those drawn upwards represent significance relative to the control. (^***^) correspond to *P* < 0.001.

The cytotoxicity of CuSO_4_ on U251 cells was initially investigated using the MTT assay. We obtained a dose dependent decrease in viability of 42 and 88% at 50 μM and 200 μM CuSO_4_ (*p* < 0.001) respectively (Supplementary Figure 1). Surprisingly cytotoxicity assessed using Trypan blue exclusion assay showed an insignificant or minimal decrease of 6% (*p* > 0.05) and 14% (*p* < 0.05) in viability of U251 cells treated with 50 and 200 μM respectively (Figure 2).

On the other hand, PA, at 250 μM, exerted a minimal effect on the viability of U251 cells in MTT assay (15% cell death, *p* < 0.001) and an insignificant effect in Trypan blue exclusion test (10% cell death, *p* > 0.05), while co-treatment with Cu-PA exhibited no protective effect, but led to a significant decrease in the viability by 81% (*p* < 0.001) and 83% (*p* < 0.001) in both tests respectively (Figure 2).

### Membrane integrity, cell morphology, and mitochondrial membrane potential in U251

**Figure 3:**
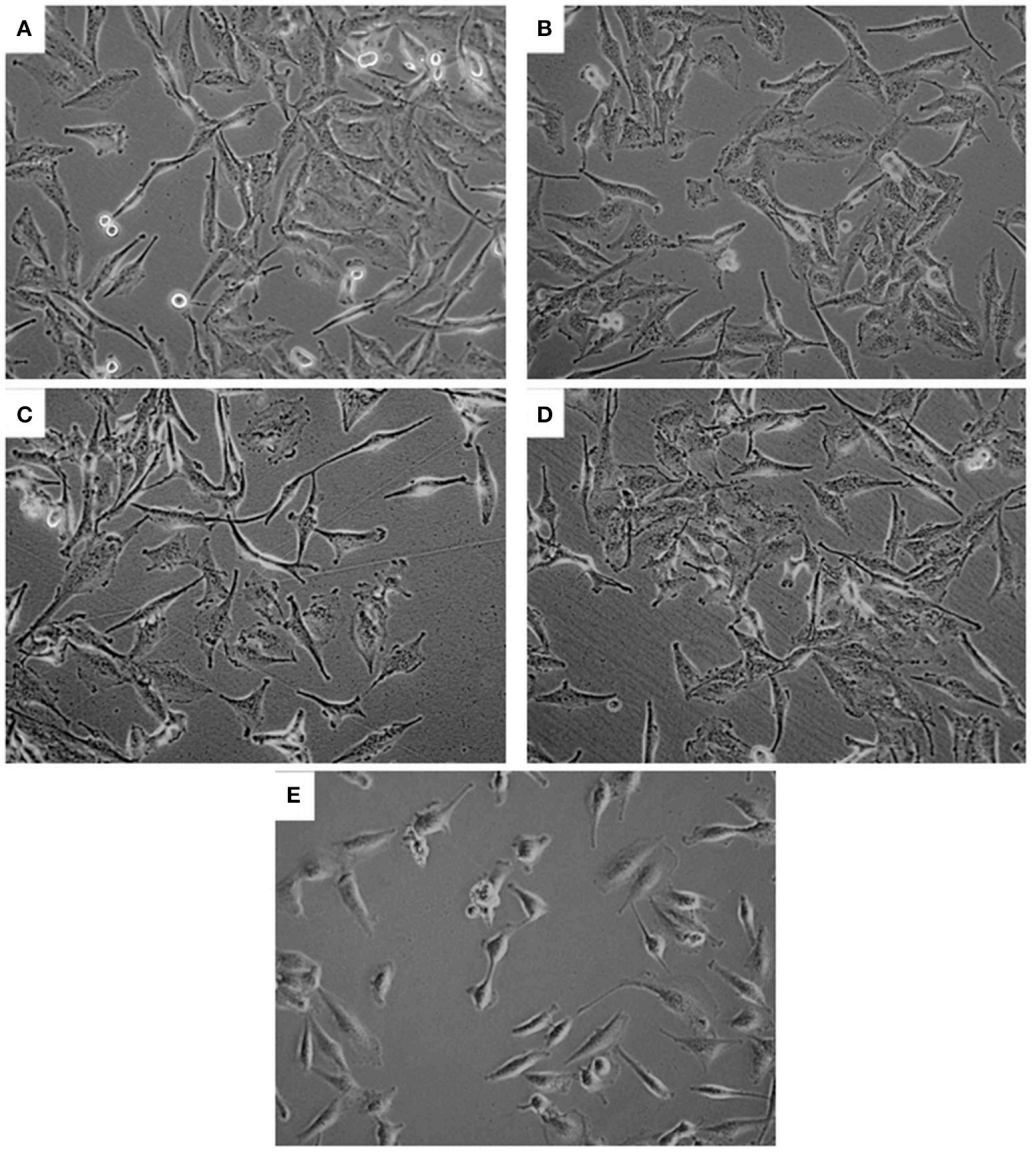
**Effect of treatment on morphology of U251 cells**. The effect of **(A)** vehicle-water (control), **(B)** 50 μM CuSO_4_, **(C)** 200 μM CuSO_4_, **(D)** 250 μM PA, and **(E)** Cu-PA on morphology of U251 cells (0.8 × 10^6^) seeded in petri dishes and treated for 24 h. Cells were examined under light microscope at 40X magnification.

No significant LDH release was noted in PA and/or CuSO_4_ treated cells, indicating no effect on membrane integrity of U251 cells (data not shown). Further examination of U251 cells treated with CuSO_4_ (50 and 200 μM) or PA (250 μM) showed no variation in the elongated morphology of the cells (Figures 3B–D) compared to untreated control (Figure 3A). However, co-treatment with Cu-PA resulted in significant morphological changes in U251 cells assuming more of a rounded morphology, indicating the initiation of apoptotic or necrotic events (Figure 3E).

**Figure 4:**
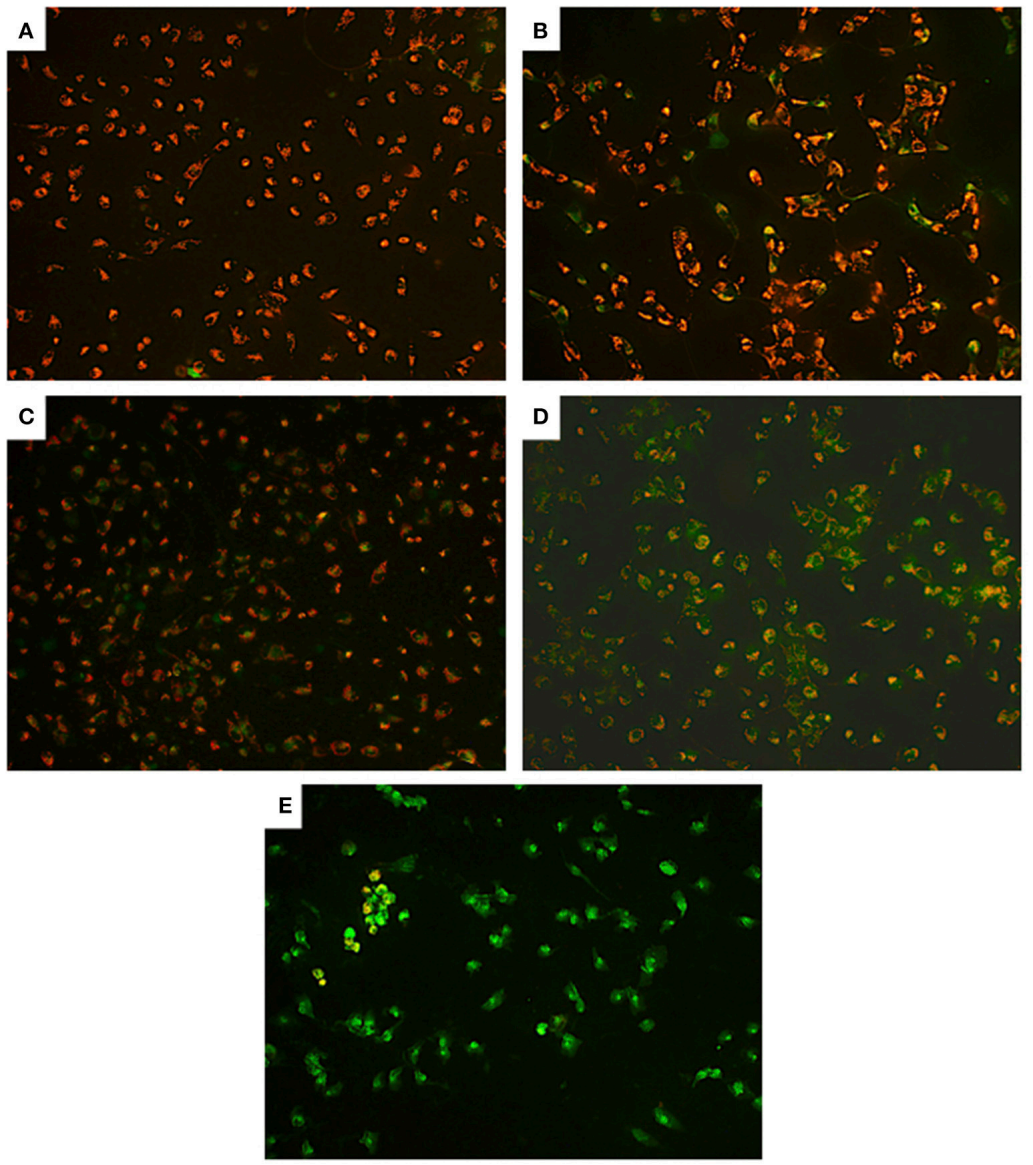
**Effect of CuSO_4_ and/or PA on mitochondrial membrane potential of U251 cells**. Mitochondrial membrane depolarization was detected using Mito PT JC-1 assay. The positively charged fluorescent probe JC-1 permeates the membrane of coupled non-apoptotic mitochondria that is negatively charged on the matrix side yielding an orange red fluorescent aggregate. In compromised uncoupled mitochondria, JC-1 get dispersed throughout the cell forming a green monomer. Representative images are for **(A)** control untreated, **(B)** 50 μM CuSO_4_, **(C)** 200 μM CuSO_4_, **(D)** 250 μM PA, and **(E)** Cu-PA, treated U251 cells.

Examining further, possible alterations at the mitochondrial level, compared to control cells (Figure 4A) we observed partial, we observed partial depolarization in all PA-treated cells (Figure 4D) but only in some of the Cu-treated U251 cells (Figures 4B,C). However, we detected complete depolarization of the mitochondrial membrane in all Cu-PA co-treated cells (Figure 4E). Compared to Cu treated U251 cells (Supplementary Figure 3) a significant round morphologic changes occurred in cells treated for 15 h with Cu-PA (Supplementary Figure 4).

### Cell cycle analysis of treated U251 cells

**Figure 5:**
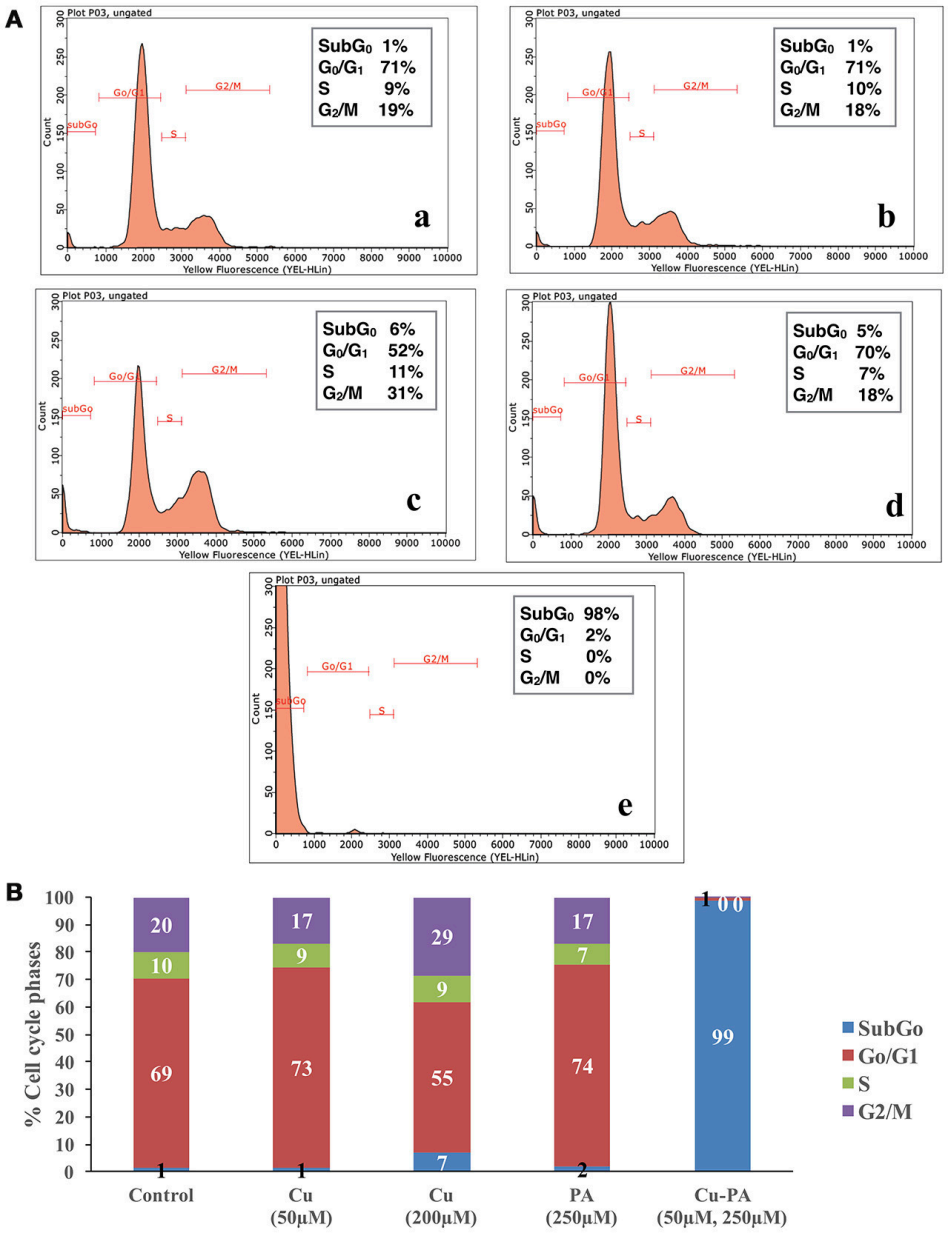
**Cell cycle analysis of U251 cells treated with CuSO_4_ and/or PA using Guava EasyCyte8 Flow Cytometer**. Figure 6A is representative image of one of three experiments: showing the cell cycle phases of U251 cells treated with: **(A-a)** vehicle water **(A-b)** 50 μM CuSO_4_, **(A-c)** 200 μM CuSO_4_, **(A-d)** 250 μM PA, and **(A-e)** Cu-PA. **(B)** is the average of 3 different experiments showing the distribution in the cell cycle phases: subG0,G0/G1, S, and G2/M.

Figure 5 shows the average distribution of Cu and/or PA treated cells in the subG0, G0/G1, S, and G2/M cell cycle phases. No remarkable changes were obtained in cell cycle distribution of Cu (50 μM), or PA (250 μM) treated U251 cells, (Figures 5A-b,d). A minimal increase was obtained in subG0 (5%) and G2 M (8%) phases in Cu (200 μM) treated cells (Figure 5A-c). However, the co-treatment of Cu-PA resulted in 98% increase in the subG0 (Figure 5B), indicating significant cell death (Figure 5A-e).

### Oxidative stress and apoptosis in Cu and/or PA treated U251 cells

**Figure 6:**
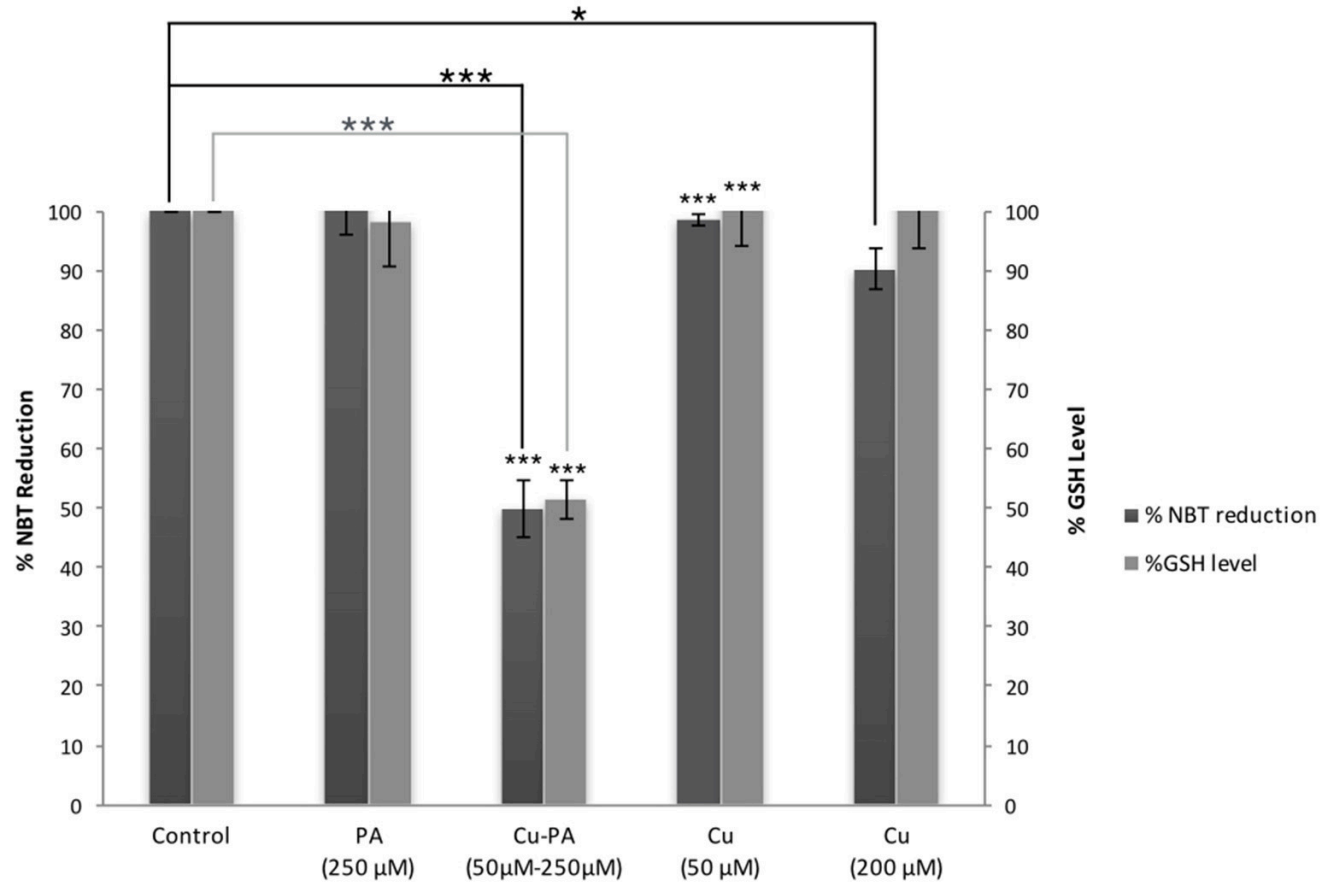
**Effect of CuSO_4_ and/or PA treated U251 cells on ROS and GSH levels**. The levels of ROS generated and GSH were determined in U251 treated cells using NBT reduction assay and the fluorometric glutathione detection kit respectively. Data presented is the mean ± SEM of 9-determinations from 3-different experiments for ROS and of 6-determinations from 3-different experiments for GSH levels. Asterisks on bars represent inter-categorical statistical significance (each category with the preceding one), and those drawn upwards represent significance relative to the control. (^*^) and (^***^) correspond to *P* < 0.05 and 0.001 respectively.

Figure 6 shows no significant effect on ROS generation or alteration in GSH level following 24-h treatment of U251 cells with Cu (50 and 200 μM) and PA (250 μM) compared to their respective control of untreated cells. However, co-treatment of U251 cells with Cu-PA (50 and 250 μM) decreased NBT reduction by 50%, indicating thus an increase of 50% in ROS level (*p* < 0.001) concomitant with a decrease in GSH level of 49% (*p* < 0.001) characteristic of early stage apoptosis.

**Figure 7:**
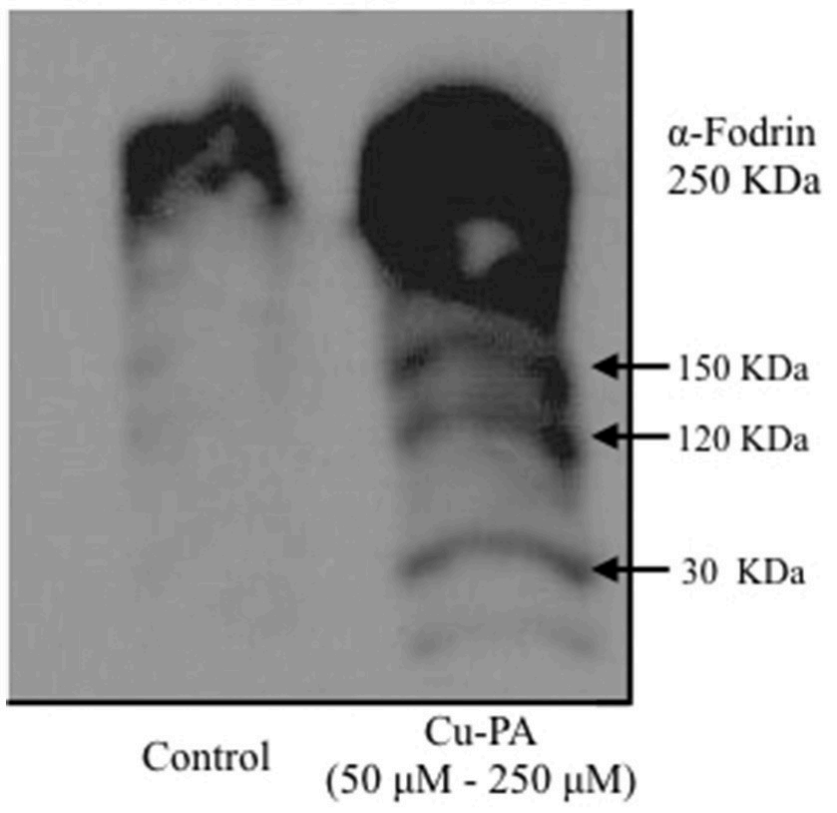
**Western blot analysis of alpha fodrin cleavage in Cu, and/or PA treated U251 cells**. Representative SDS-PAGE image of U251 protein lysate (75 μg) Cu-PA treated clearly shows the 150 KDa band indicative of proteolytic cleavage and characteristic of apoptosis. No cleavage products were detected in Cu or PA treated cells (Supplementary Figure 2).

To further confirm apoptosis and rule out necrosis, cleavage of α-fodrin, an apoptotic marker was examined in Cu and/or PA treated cells using western blot analysis. No fragmentation was detected in U251 cells treated with PA or Cu alone (Supplementary Figure 2); however, in Cu-PA co-treated U251 cells, α-Fodrin cleavage was induced resulting in many bands detected at different KDa (Figure 7).

### Protective effect of antioxidants on Cu-PA treated U251 cells

**Figure 8:**
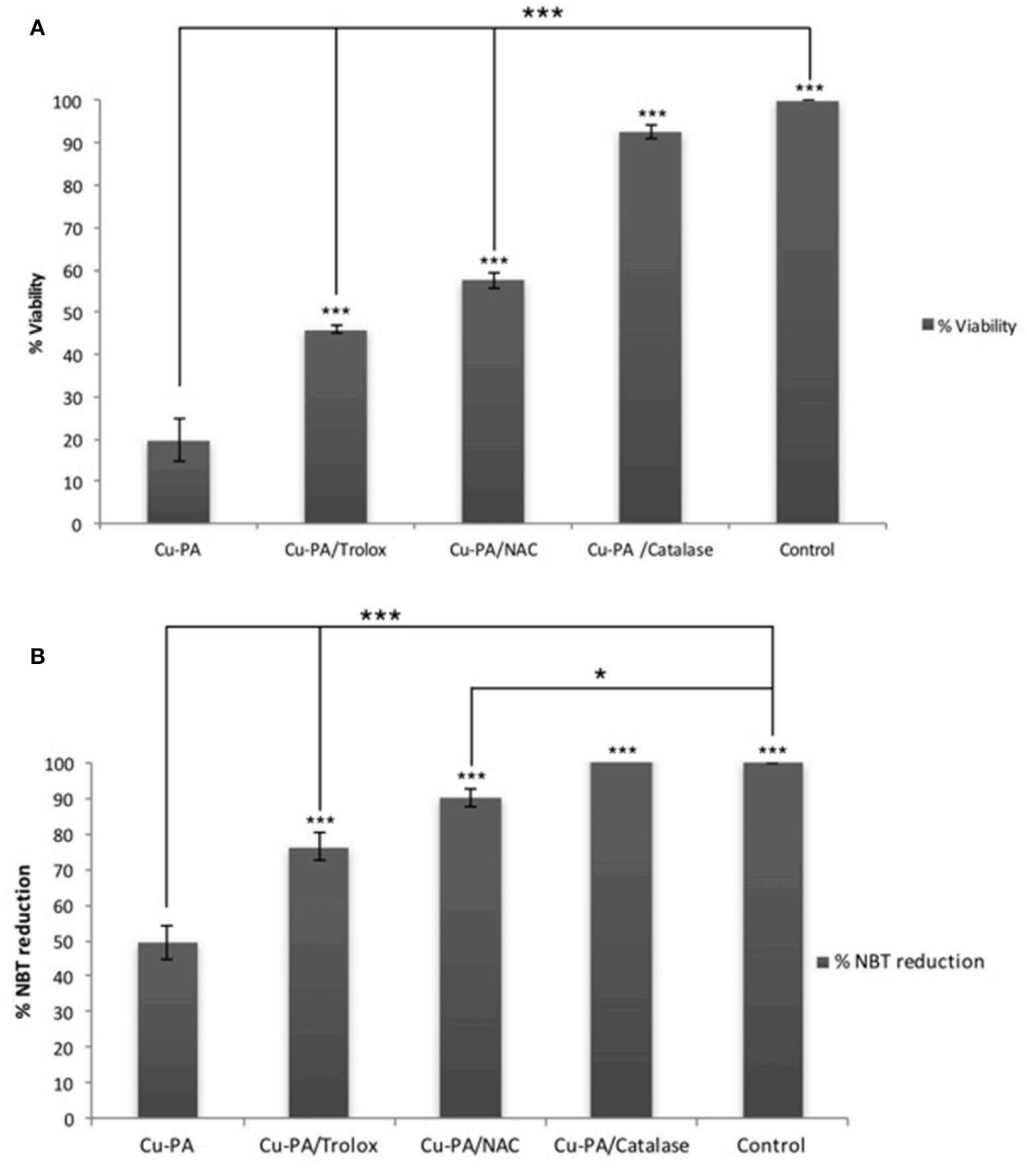
**Protective effect of antioxidants: NAC (5 mM), Trolox (100 μM), and catalase (500U) on: (A)** viability and **(B)** ROS generation in Cu-PA treated U251 cells. Viability was determined using MTT cell proliferation assay, whereas level of ROS generated was determined using NBT reduction assay. Data presented is the mean ± SEM of 6-determinations from 3-different experiments. Asterisks on bars represent inter-categorical statistical significance with Cu-PA category, and those drawn upwards represent significance relative to the control. (^*^) and (^***^) correspond to *P* < 0.05 and 0.001 respectively.

**Figure 9:**
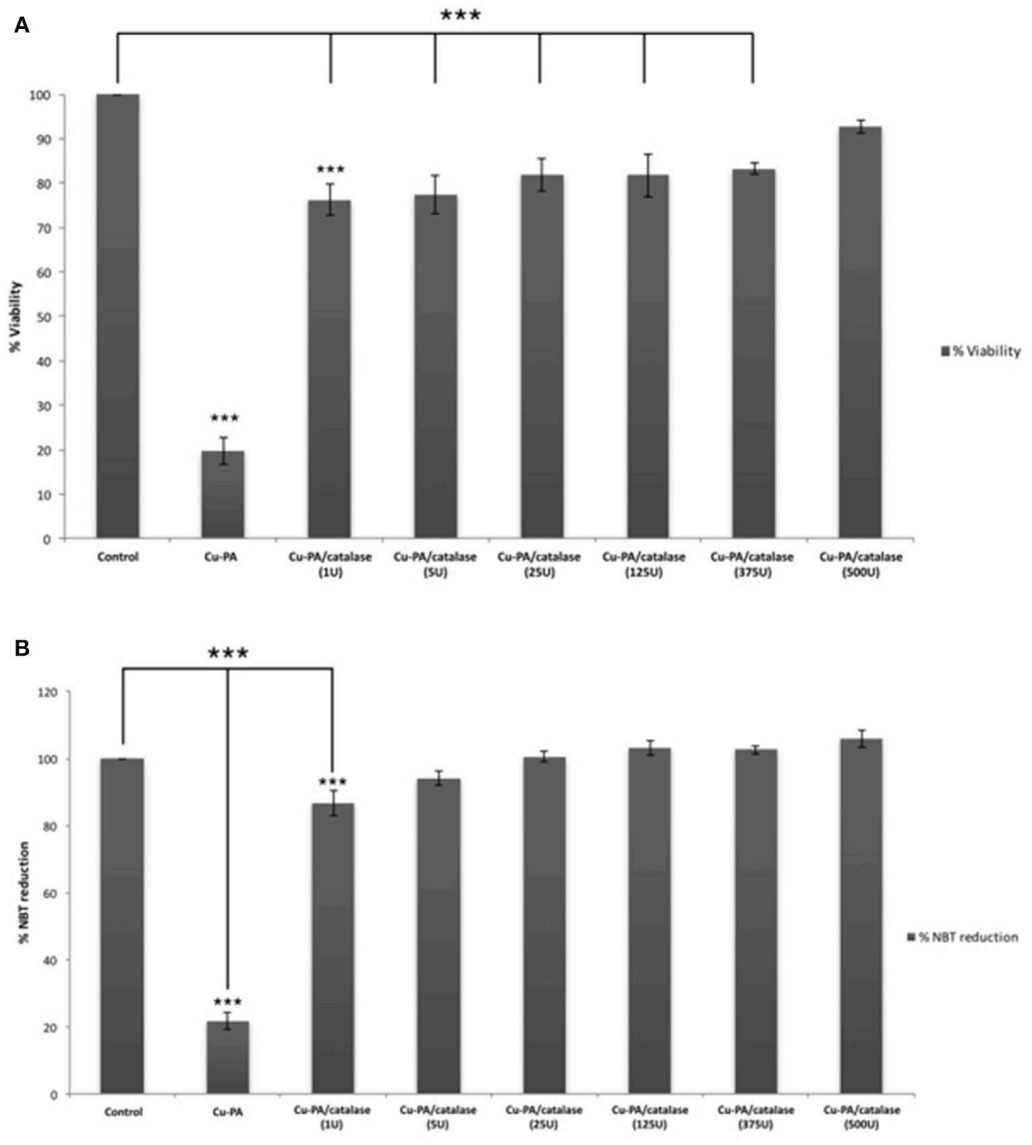
**Dose-dependent effect of catalase enzyme on viability (A)** and ROS generation **(B)** in Cu-PA treated U251 cells. Viability was assessed by MTT cell proliferation assay, whereas ROS generation was determined by NBT reduction assay. Data presented is the mean ± SEM of 6-determinations from 3-different experiments. Asterisks on bars represent inter-categorical statistical significance (each category with the preceding one), and those drawn upwards represent significance relative to the control. (^***^) correspond to *P* < 0.001.

Figure 8 shows the protective effect of antioxidants (NAC, Trolox) and catalase enzyme on Cu-PA induced toxicity on U251 cells. Pretreatment of U251 cells with NAC (5 mM), or Trolox (100 μM) for 2 h, restored partially the viability of Cu-PA treated cells by 38% (*p* < 0.001) and 26% (*p* < 0.001), respectively; whereas co-treatment with catalase (500U) restored the viability of Cu-PA treated cells by 73% (*p* < 0.001; Figure 8A). Similarly, pretreatment with NAC and Trolox decreased the generation of ROS by 40% (*p* < 0.001) and 27% (*p* < 0.001), respectively; whereas co-treatment of catalase completely restored the oxidative stress (50% decrease in ROS, *p* < 0.001; Figure 8B). When different concentrations of catalase enzyme (1–500U) were co-treated with Cu-PA on U251 cells, no significant difference was observed in their protective effect on either viability (Figure 9A) or ROS generation (Figure 9B) indicating that the reduction of hydrogen peroxide by catalase is protective to the Cu-PA co-treatment.

### Expression of copper-binding ceruloplasmin in U251 cells

**Figure 10:**
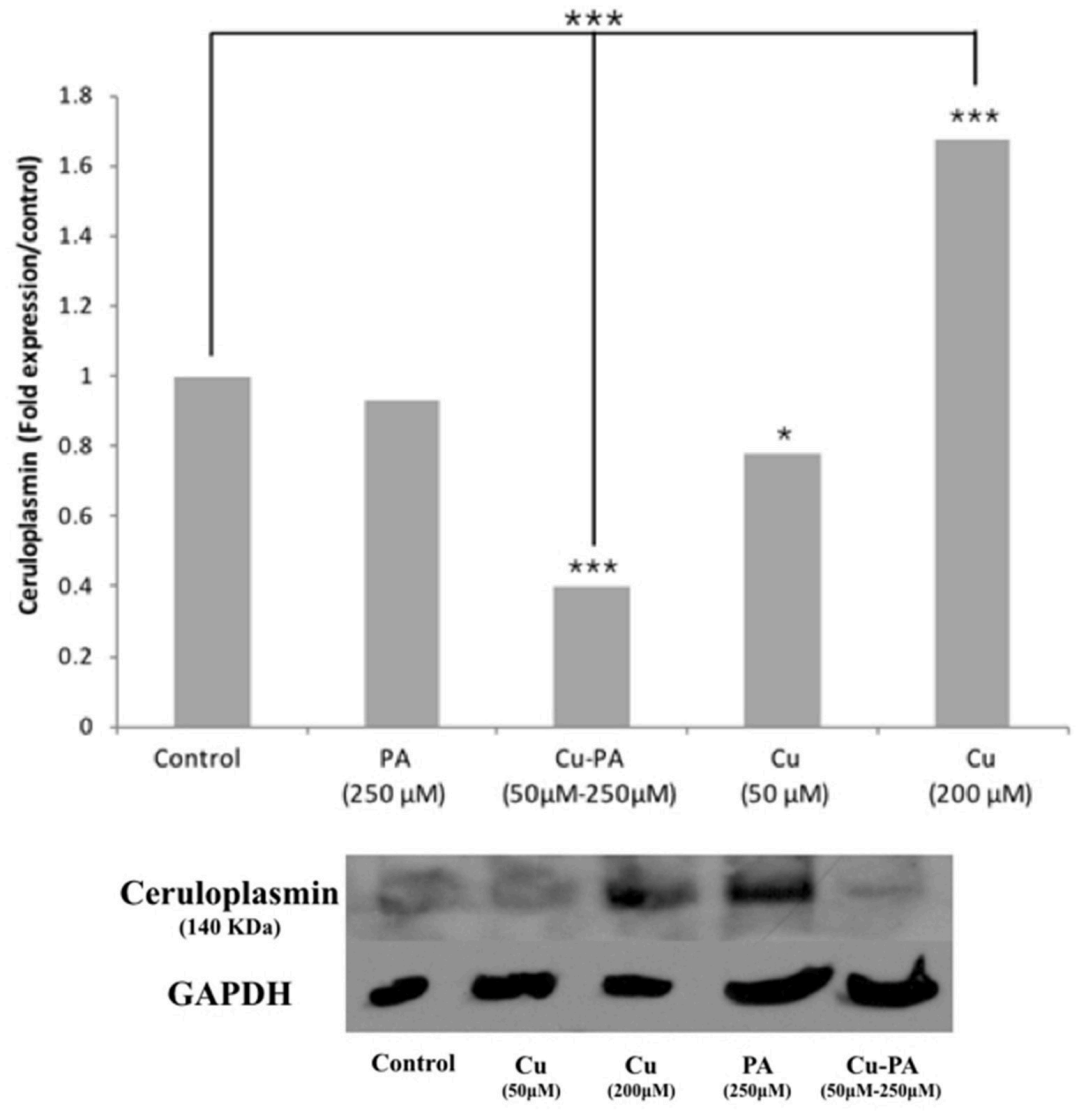
**Effect of CuSO_4_ and/or PA treated U251 cells on ceruloplasmin expression level**. Ceruloplasmin expression was determined using western blot analysis on protein lysates of different cell treatments. Fold expression was determined relative to the control following quantitative assessment of bands using Image J. Expression level of treated and control samples normalized to their respective GAPDH. Asterisks on bars represent inter-categorical statistical significance (each category with the preceding one), and those drawn upwards represent significance relative to the control. (^*^), and (^***^) correspond to *P* < 0.05, 0.01, and 0.001 respectively. Image is representative of 2 experiments.

Compared to control, neither Cu (50 μM) nor PA (250 μM) exerted any effect on the Cp expression level. However, when cells were treated with Cu-PA, a significant decrease in Cp expression level by 0.6-fold (*p* < 0.001) was obtained. The response of the cells to Cu treatment (200 μM) was confirmed by the increase (0.67-fold increase, *p* < 0.001) in Cp expression (Figure 10).

### Effect of CuSO_4_ and/or PA on neuronal PC12 cells

**Figure 11:**
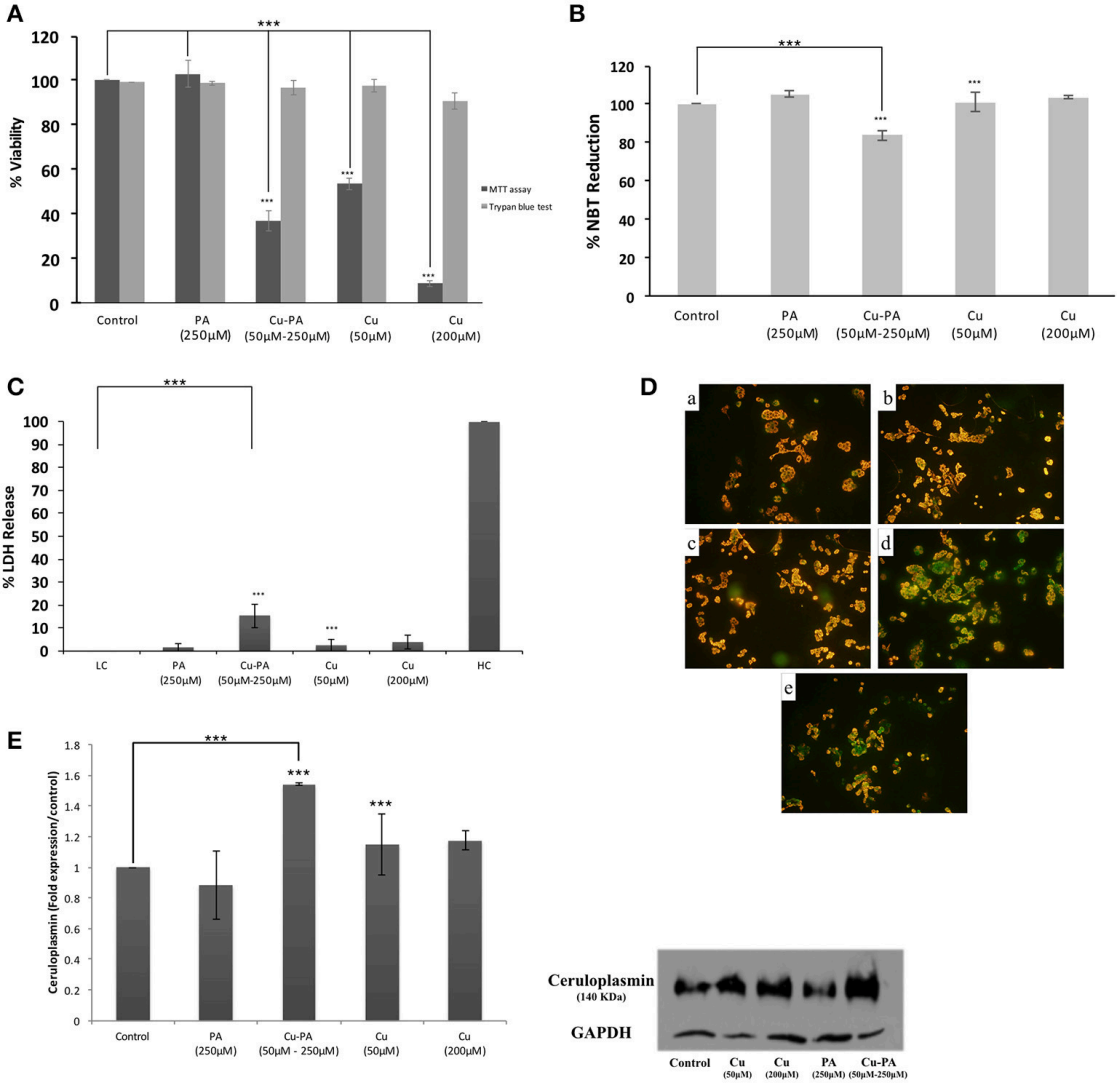
**Effect of CuSO_4_ and/or PA treated PC12 cells on: (A)** viability using MTT assay and Trypan blue exclusion test, **(B)** ROS level using NBT reduction assay, **(C)** LDH release using cytotoxicity detection kit, **(D)** mitochondrial membrane potential using Mito PT-JC1 assay (a) control; (b) 50 μM CuSO_4_; (c) 200 μM CuSO_4_; (d) 250 μM PA; and (e) Cu-PA, treated PC12 cells, and **(E)** Cp expression level using western blot analysis. Data presented is the mean ± SEM of: 15-determinations from 5-different MTT experiments and 3-determinations from 3-different Trypan blue exclusion experiments; 12-determinations each from 4-different NBT reduction experiments and LDH release assays and of 3-determinations from 3-different Western Blot analysis. Asterisks on bars represent inter-categorical statistical significance relative to a control. (^***^) correspond to *P* < 0.001.

Similar viability profile to that of U251 was obtained in PC12 cells treated with Cu or PA. However, Cu-PA co-treatment exerted a different effect on PC12: no significant decrease in viability was obtained by Trypan blue exclusion test, whereas 63% decrease (*p* < 0.001) was reported using MTT assay (Figure 11A).

PA or Cu treatment exerted no effect on ROS generation (Figure 11B), LDH release (Figure 11C), GSH level, cell morphology, and cell cycle phases (data not shown). On the other hand, the co-treatment slightly increased ROS generation (Figure 11B) and LDH release (Figure 11C) by 16% each (*p* < 0.001), increased subG0 phase by 12%, but did not result in any morphological change (Supplementary Figure 5) or variation in GSH level. Furthermore, neither Cu nor PA exerted any effect on the Cp expression level; however, Cu-PA co-treatment increased (Figure 11E) the Cp expression (*p* < 0.001). At the mitochondrial level, partial depolarization of mitochondria was induced by PA and Cu-PA while no effect was observed in Cu treated cells (Figure 11D).

### Effect of CuSO_4_ and/or PA on SH-SY5Y cells

We have also examined the effects of Cu and/or PA on another neuronal cell line namely the neuroblastoma SH-SY5Y cells. Morphological changes were observed with PA and Cu-PA treated cells (Supplementary Figure 6). Similar to U251 cells, the viability of SH-SY5Y cells decreased significantly (82%) following co-treatment with Cu-PA. However, unlike U251 and PC12 cells, we obtained a significant decrease in viability of 20 and 40% with Cu and PA treatment, respectively. Further investigations on the effect of treatments on ROS level exhibited a different profile from those obtained with U251 and PC12 cells. Treatment of SH-5H5Y with Cu or PA caused a decrease in NBT reduction of 28 and 34% respectively indicating that these cells are more sensitive to PA treatment alone, unlike U251 and PC12; co-treatment with Cu-PA resulted in 41% decrease in NBT reduction (Supplementary Figures 7A, B).

## Discussion

D-Penicillamine (PA) is a commonly used drug to treat WD, as well as heavy metal poisoning, cysteinuria, rheumatoid arthritis, primary biliary cirrhosis, and progressive systemic sclerosis (Naik et al., 2010). However, the side effects of PA treatment are numerous. The main concern is the worsening in neurologically presenting patients, who do not recover even with PA discontinuation (Brewer et al., 1987; Kalita et al., 2014). Neurologic deterioration has been reported in 50% of WD patients with neurologic presentation. Many have recommended avoiding prescription of PA when less harmful drugs are available, such as zinc and trientine. In Lebanon, both zinc and trientine, if available, are quite expensive leaving clinicians with one option, PA prescription.

Therefore, this study was undertaken to explore the safety of this de-coppering drug; more specifically it aimed at investigating the *in vitro* effect of PA and Cu on neural cell lines. To mimic the condition of WD patients, cells were treated for 24 h with varying concentrations with Cu, PA or both.

The choice of PA concentration (250 μM), was not arbitrary, but based on previous studies, on human leukemia and breast cancer cells, reporting non-toxicity of PA at 200–500 μM (Gupte and Mumper, 2007a,b). Likewise, the Cu concentration used was chosen relative to reported approximate range of physiological Cu concentrations (70 μM and 100 μM) in cerebrospinal fluid and synaptic cleft respectively (Kardos et al., 1989; Opazo et al., 2014).

Initially, MTT assay was used to determine the IC50 (50 μM) in Cu-treated U251 cells (Supplementary Figure 1). However, MTT viability results were discordant with those using trypan blue exclusion assay (Figure 2). While the latter is a qualitative assay determining the number of viable cells based on the cell membrane integrity (Strober, 2001), the MTT assay measures mitochondrial function by monitoring the effect of Cu, on the activity of mitochondrial dehydrogenase (van Meerloo et al., 2011). Thus, this discordance may be attributed to the possible interference of Cu with the MTT assay. Previous *in vivo* and *in vitro* studies have reported inhibition of the activity of the mitochondrial NADH-dependent dehydrogenases by Cu without leading to cell death (Sheline and Choi, 2004). No major effect on the viability of PA treated U251 cells was obtained using either assay. Co–treatment however, with Cu-PA (50–250 μM) decreased significantly and to the same extent the viability in either assay, suggesting sensitivity of U251 cells to Cu-PA co-treatment, but not to Cu or PA alone.

Our findings are similar to previous studies which reported: (a) small decrease in cell proliferation of cultured rabbit articular chondrocytes treated with either CuSO_4_ or D-PA but marked increase in the extent of growth inhibition of cells treated with D-PA in association with copper (Clain et al., 1988); and (b) cytotoxicity of Cu-PA (10 ≤ 400 μM) on human breast cancer MCF-7 and BT474 cells, and human leukemia HL-60 cells (Gupte and Mumper, 2007a).

Consistent with the obtained results, we observed no morphological changes in PA or Cu treated cells. However, co-treatment with Cu-PA induced the loss of the distinctive elongated morphology of U251 cells into rounded shape. For further assessment of the morphological changes and membrane derangement, we examined whether necrosis occurs following the different treatments by measuring the LDH level released (Vairetti et al., 2005; Chan et al., 2013). In our study, no significant release of LDH into the media of cultured cells was detected in any of treatments. These findings are in congruence with those reporting 10% LDH release from PC12 cells treated for 48 h with 500 μM Cu (10-fold the concentration used in the current study) (Belyaeva et al., 2012).

Copper (Cu) is an important component of electron transport chain protein complexes, such as cytochrome c oxidase (Horn and Barrientos, 2008). Previous study had reported impaired mitochondrial enzymatic activities in ATP7B^−/−^ animals with apparent clinical manifestations of WD. In addition, isolated liver mitochondria treated with Cu chelators showed structural and functional changes (Zischka et al., 2011). Although intracellular level of copper was not determined in this study, mobilization of intracellular copper sites of mitochondria would perturb copper level and derange mitochondrial function (Bush, 2000). Qualitative assessment of the coupling state of the mitochondrial membrane potential (Mito JC-1 kit) showed no effect on membrane potential following treatment of U251 cells with Cu while PA induced partial depolarization. However, the co-treatment with Cu-PA resulted in complete depolarization. This suggests that dissipation of the mitochondrial membrane potential is not resulting solely from the possibility of Cu mobilization induced by PA, but from a possible toxic intermediate or component derived or resulted from PA interaction with Cu.

Mitochondrial dysfunction may induce oxidative stress that results from generation of ROS and/or depletion of GSH (Gyulkhandanyan et al., 2003). In this study, we obtained no significant variation in ROS or GSH level in Cu or PA treated U251 cells, in concordance with a previous study on astrocytes reporting that copper exerted no significant changes in ROS and GSH levels and mitochondrial membrane potential (Gyulkhandanyan et al., 2003; Scheiber and Dringen, 2011). Having stronger anti-oxidative potential than neurons, astrocytes may protect them from compound induced oxidative stress (Desagher et al., 1996; Scheiber and Dringen, 2011; Dringen et al., 2013). However, increased oxidative damage in neuronal P19 and PC12 cells occurred at a Cu concentration greater than 0.5 mM (Jazvinscak Jembrek et al., 2014). On the other hand, the obtained depletion in GSH level in U251 cells co-treated with Cu-PA, is in line with generated ROS and depolarization of mitochondrial membrane potential. In one study, ROS production was insignificant with either PA or CuSO_4_ treated leukemia/breast cancer cells; while co-treatment with Cu-PA exhibited a concentration-dependent H_2_O_2_-mediated cytotoxicity with a concomitant decrease in intracellular GSH levels (Gupte and Mumper, 2007a). Impairment in cellular defense mechanisms against ROS causes oxidative stress induced neurodegenerative disorders. These include endogenous enzymes (Superoxide dismutase, Glutathione peroxidase), Low molecular weight antioxidants (glutathione, tocopherols, ascorbic acid, Vitamin A, lipoic acid) and precursors of endogenous antioxidants (NAC) (Gilgun-Sherki et al., 2001). Previous studies reported the considerable depletion ascorbic acid level and compromised antioxidant status in plasma of untreated WD patients (Ogihara et al., 1995; Attri et al., 2006). Several *In vivo* studies have alluded to the protective effects of the antioxidants, such as lipoic acid (Yamamoto et al., 2001), Vitamin E (Yamazaki et al., 1993; Fryer, 2009), ascorbic acid and thioredoxin (Hawkins et al., 1995) against Cu-induced hepatitis in Long Evan Cinnamon rats an animal model of WD. Other *in vitro* studies demonstrated the biochemical role of mitochondrial enzyme cofactors in attenuating copper induced death (Sheline et al., 2002); for instance the beneficial role of thiamine supplementation has been widely reviewed and documented (Luong and Nguyen, 2013). In addition the decrease in GSH and protein thiol levels was reported to enhance the pro-apoptotic conditions and oxidative stress in hepatocytes and neurons of WD rodent model (Samuele et al., 2005).

**Figure 12:**
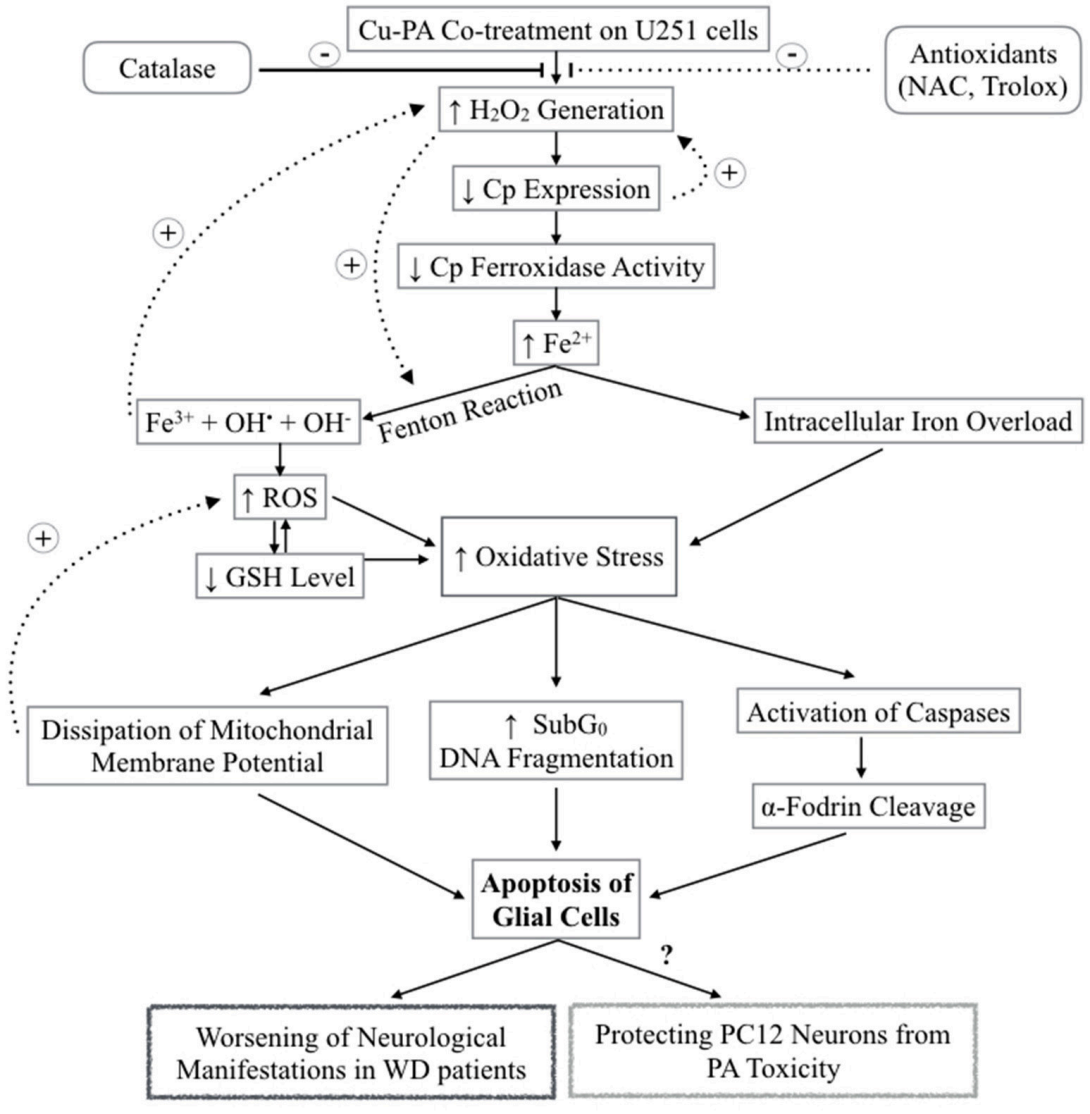
**Schematic Diagram summarizing the effect of Cu-PA treatment on neural cells H_2_O_2_ generation increases following Cu-PA treatment on U251 Glial cells**. Entry of H_2_O_2_ into the cells increases oxidative stress intracellularly. Alternatively, it mobilizes intracellular copper leading to a decrease in CP expression. Decrease in level of incorporated Cp copper would decrease ferroxidase activity involved in iron loading onto iron binding/transporting proteins. The increase in Fe^3+^ in presence of H_2_O_2_ generates hydroxyl radicals and increases ROS level leading to a decrease in GSH level. Consequences of the increased oxidative stress are: dissipation of mitochondrial membrane potential that triggers apoptotic events; increase in SubG0 phase /DNA fragmentation; and activation of caspases causing proteolytic cleavage of α-fodrin eventually leading to cell death of U251 glial cells. Catalase enzyme protected completely treated U251 cells whereas NAC and Trolox were partially protective.

Starkebaum and Root (1985) reported that during chelation, reduction of Cu^2+^–Cu^+^ generates PA (thiyl) radicals. Regeneration of Cu^2+^occurs at the expense of the consecutive reduction of oxygen by Cu^+^ into superoxide (${\text{O}}_{2}^{.-}$) radical, and then hydrogen peroxide (H_2_O_2_). Figure 12 presents the different steps leading to the net PA oxidation that results in H_2_O_2_ generation (Figure 12), at a molar ratio of 2:1 (Starkebaum and Root, 1985). To examine whether cytotoxicity of Cu-PA is mediated by H_2_O_2_ we studied the potential role of antioxidants (NAC, Trolox, and catalase) in reversing Cu-PA toxicity. Co-treatment with catalase, a key antioxidant enzyme specifically responsible for decomposition of H_2_O_2_ (Marklund et al., 1982), completely restored the viability and decreased oxidative stress level in Cu-PA co-treated cells. However, NAC and Trolox were less protective than catalase. These results confirm that H_2_O_2_ is the culprit molecule mediating oxidative stress and apoptosis in Cu-PA treated cells.

H_2_O_2_ is a major oxidant known to cause neuronal injury (Coombes et al., 2011). The increase in oxidative stress alters functions of glial cells contributing thus to the pathogenesis of neurodegenerative diseases as well as to the selective vulnerability of neurons (Dringen, 2000). One biochemical marker of oxidative stress is the level of GSH, the decrease of which was shown to trigger apoptotic events (Franco and Cidlowski, 2009) that can be verified using flow cytometry. Comparing % distribution of cell cycle phases between control and PA or Cu treated U251 cells, we found no significant difference, confirming the attribution of the decrease in cell viability of Cu-treated cells (MTT assay) to a decrease in the dehydrogenase activity. However, U251 cells co-treated with Cu-PA caused a 99% increase in subG0 phase, compared to 1% in control cells, indicating DNA fragmentation (Sedlak et al., 1999) leading to cell death by apoptosis or necrosis (Higuchi, 2003). The depletion of GSH level and the absence of LDH release in Cu-PA treated U251 cells supports apoptosis rather than necrosis. A hallmark of apoptosis includes the activation of proteases that cleave structural proteins, initiating a cascade of events. More specifically the proteolysis of α-fodrin by caspase-3 and calpains contributes to structural rearrangements including blebbing during apoptosis (Martin et al., 1995; Janicke et al., 1998; Kahaly et al., 2005). Using western blot analysis, we identified a 150 KDa fragment, the calpain-dependent cleavage of α-fodrin in Cu-PA co-treated U251 cells (Supplementary Figure 2).

Ceruloplasmin, the main transporter of copper in blood, is an acute phase reactant protein, the serum concentration of which increases during inflammation or trauma (Gitlin, 1988). It is predominantly synthesized in the liver and is also expressed in various tissues including spleen, lungs, and brain (Aldred et al., 1987). Cp is expressed in 2 isoforms as a secreted form or Glycosylphosphatidylinositol-Cp anchored to plasma membrane (Patel et al., 2000). In the current study, we reported that Cp expression increased by 67% in Cu-treated U251, consistent with the ability of astrocytes to recognize, transport and store nutrients (Scheiber et al., 2014). Treatment with PA exerted no effect; whereas co-treatment with Cu-PA decreased its expression by 60%. Since similar results were not obtained with PA treatment alone, the decrease of Cp expression by Cu-PA may not be due to mobilization of intracellular Cu stores. Instead, this could be the result of Cu-catalyzed PA oxidation generating extracellular H_2_O_2_ that has been reported to decrease the Cp level through an mRNA decay mechanism (Tapryal et al., 2009). This poses a question regarding the other consequences of the lifelong management of WD patients using copper chelators. Cp belongs to multi-copper oxidase family (Cha and Kim, 1999) with ferroxidase activity implicated in iron loading onto serum transferrin, as well as in iron homeostasis (Okamoto et al., 1996). One possible consequence of Cu chelation is impairment in iron loading which is copper-Cp dependent preventing the oxidation of Fe^2+^ to Fe^3+^ leading ultimately to iron deposition. The piled Fe^2+^ in the presence of the Cu-PA induced generated H_2_O_2_ will produce hydroxyl and superoxide radicals through fenton reactions eventually leading to cell death (Agil et al., 1995).

To examine whether the effects of Cu and/or PA are specific to U251 cells only, we used PC12 cells that are widely accepted as model system for: neuronal differentiation, neurosecretion, neuronal injury (Pera et al., 2013). Furthermore, PC12 (differentiated and undifferentiated) were used to assess neurotoxicity underlying degenerative disorders, following their exposure to necrotic insult (Jiang et al., 2014), as well as apoptosis during brain injury (Ballesteros et al., 2007; Minambres et al., 2008). In a recent report, PC12 were also used as a model to study Cu metabolism during PC12 differentiation into neurons (Ogra et al., 2016).

In our study, whereas Cu or PA treated PC12 cells had similar effects to those of U251 cells, Cu-PA treated PC12 exerted a milder effect: generated ROS (16%), increase in sub G0 phase (12%), LDH–release (16%) and a partial depolarization of mitochondria. The resistance of PC12 to the co-treatment is unexplainable and may possibly be attributed to species difference, being derived from rats, PC12 cells sensitivity may differ from human U251 cells. A plausible reason may be attributed to the undifferentiated state of the PC12 cells, although they have been useful in identifying protein fragments underlying neuronal dysfunction. The effect of Cu-PA was not limited to glioblastoma U251 cells but is extended to differentiated neuroblastoma SH-SY5Y cells identifying thus neuronal cells as potential targets for Cu-PA. However, SH-SY5Y cells differed from Glioblastoma U251 cells, in: (a) being more sensitive (decreased in viability) to free copper or PA but as sensitive to Cu-PA co-treatment and (b) the level of generated ROS post Cu-PA treatment was not remarkably different from Cu or PA treated cells. Our finding are in line with a previous study reporting mitochondrial dehydrogenase inhibition in Cu induced toxicity of neuroblastoma cell line (Arciello et al., 2005). Hence, both cell lines may serve as potential target to Cu-PA. Many studies have reported the involvement of diverse neural cells including neuronal and glial cells in neurological disorders induced in patients with WD (Horoupian et al., 1988; Lewandowska et al., 2004; Merker et al., 2005).

In this study, we investigated the sensitivity of U251 cells to Cu-PA treatment at a Cu concentration below the toxic levels of Cu (500 μM). Cu-PA co-treatment triggers remarkable extracellular generation of H_2_O_2_ which permeates the plasma membrane of U251 cells causing a decrease in Cp expression by mRNA degradation, leading to a decrease in glutathione level, an increase in oxidative stress by Fenton reactions. This may initiate a cascade of events eventually leading to apoptosis, as evidenced by the dissipation of the mitochondrial membrane potential, DNA fragmentation, and cleavage of apoptotic marker α-fodrin (Figure 12).

One possible explanation for the deterioration of the neurological symptoms may be the low activity of catalase in the brain. Both gray and white matters of the human brain have immeasurable levels of catalase (Marklund et al., 1982; Baydas et al., 2002). For this reason, we propose that antioxidant supplements be part of the therapeutic regimen with PA treated WD patients with neurological presentation.

There are several limitations to our study. These include the use of human cancer cells (U251 and SH-SY5Y) which might have normal copper metabolism as well as the use of immortalized rat derived PC12 as normal cells. It is recommended that future studies addressing the effect of Cu-PA be performed *in vivo* or on isolated primary neuronal cells.

To conclude, our findings identify U251 cells with both positive (preventive) and negative (toxic) roles. Being the first target and primary site of exposure to external factors and drugs (Scheiber et al., 2014), the apoptotic cell death of the astrocytes provides a mechanism (remarkable increase in ROS) that might protect the underlying neuronal cells. MRI studies reported diffusion restriction and T2 hyperintensity in the white matter of neurologic WD patients caused from damaged glial system with compromised ability to clear oxidative stress (Kalita et al., 2014; Ranjan et al., 2015). The demonstrated toxicity in U251 and SH-SY5Y cells co-treated with Cu-PA may shed some light on one of the possible underlying mechanisms that worsens the manifestations of WD patients with neurologic manifestations. In a copper rich environment, such as the one characterizing WD, the expected response (generation of H_2_O_2_) would be exaggerated. In addition, our findings pose an important question on the consequence of PA treatment on Fe homeostasis, and possible Fe deposition, which might require new modalities in managing side effects of the lifelong treatment of WD patients.

## Author contributions

MK performed all the experiments, as part of her Master's thesis. MJ contributed partially to the experimental work and in drawing the figures. FK provided the cells, read and commented on the manuscript, while KB and JU supervised and commented on the work. MK, MJ, and JU all contributed to the write up. Grants supporting the work were given to JU. WA provided us with the SH-SY5Y cells and the conditions to grow these cells. AM performed all the experiments on this cell line supervised by WA.

### Conflict of interest statement

The authors declare that the research was conducted in the absence of any commercial or financial relationships that could be construed as a potential conflict of interest.
