# Supplementary material for: Chemosensitivity of U251 Cells to the Co-treatment of D-Penicillamine and Copper: Possible Implications on Wilson Disease Patients
Source: Front Mol Neurosci. 2017 Jan 31;10:10. doi: 10.3389/fnmol.2017.00010 (PMC5281637; doi:10.3389/fnmol.2017.00010)
Supplement: Supplementary file 1 [file DataSheet1.docx]

Supplementary Figure Legend

**Figure 1:** **Effect of CuSO_4_ on viability of U251 cells.**

The dose-dependent effect of CuSO_4_ (10-200 μM) on viability of U251 cells (0.8 x 10^4^) was determined using MTT assay. Absorbance was measured at 595 nm and presented as % viability comparing treated to control of untreated cells. Data presented is the mean ± SEM of 18-determinations from 6-different experiments. Asterisks on bars represent inter-categorical statistical significance (each category with the preceding one), and those drawn upwards represent significance relative to the control. (*), (**), and (***) correspond to P<0.05, 0.01, and 0.001 respectively.

**Figure 2:** **Western blot analysis of alpha-fodrin cleavage in Cu, and /or PA treated U251 cells.**

(30 µg) Cu, PA and Cu-PA treated cells. The Cu-PA treated cells clearly show the 150 KDa band indicative of proteolytic cleavage and characteristic of apoptosis.

**Figures 3 and 4: Supplementary images of U251 Cu-treated (Fig. 6) and Cu-PA-treated (Fig. 7) cells that were visualized at 20x magnification after 15 hours of treatment.**

Round morphology of Cu-PA treated cells is significantly different from Cu-treated cells.

**Figure 5: Effect of treatment on morphology of PC12 cells.**

The effect of **A)** vehicle-water (control), **B)** Cu-PA (50 μM, 250 μM) on morphology of PC12 cells (0.8 x 10^6^) seeded in petri dishes and treated for 24hours. Cells were examined under light microscope at 40X magnification.

**Figure 6: Effect of treatment on morphology of SH-SY5Y cells.**

The effect of **A)** vehicle-water (control), **B)** 50 μM CuSO_4_, **C)** 250 μM PA, and **D)** Cu-PA on morphology of SH-SY5Y cells (100,000) seeded in petri dishes and treated for 24hours. Cells were examined under AxioVert A1 at 20X magnification.

**Figure 7: Comparative effect of Cu and/ or PA on viability of and ROS generation in SH-SY5Y cells.**

**A)** SH-SY5Y cells (1x10^5^/well) were treated with CuSO_4_ (50 μM), PA (250 μM) or Cu-PA (50 μM - 250 μM) for 24 hours. Data presented as % viability is the mean ± SEM of 6-determinations using trypan blue exclusion assay. **B)** The levels of ROS generated was determined in SH-SY5Y (5x10^3^ cells/well) seeded in 96-well plate then treated cells using NBT reduction assay. Data presented is the mean ± SEM of 5-determinations from 3-different experiments.
