# Supplementary figures and images for: Chemosensitivity of U251 Cells to the Co-treatment of D-Penicillamine and Copper: Possible Implications on Wilson Disease Patients
Source: Front Mol Neurosci. 2017 Jan 31;10:10. doi: 10.3389/fnmol.2017.00010 (PMC5281637; doi:10.3389/fnmol.2017.00010)

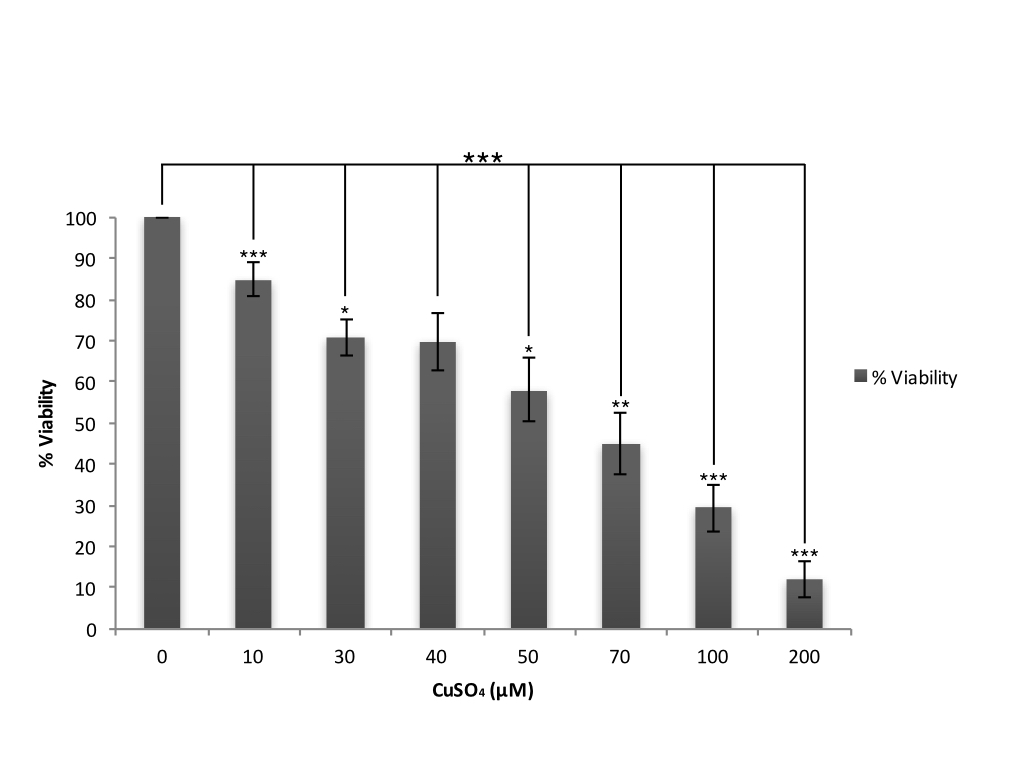

Supplement: Supplementary file 4 [file Image1.JPEG]

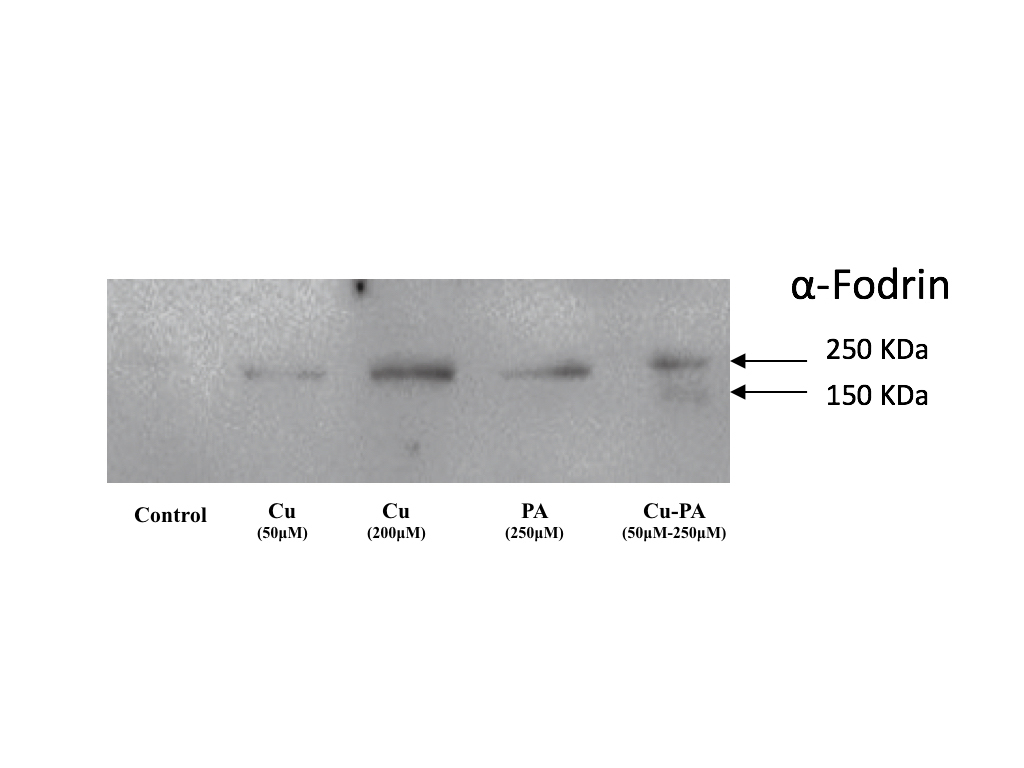

Supplement: Supplementary file 5 [file Image2.jpg]

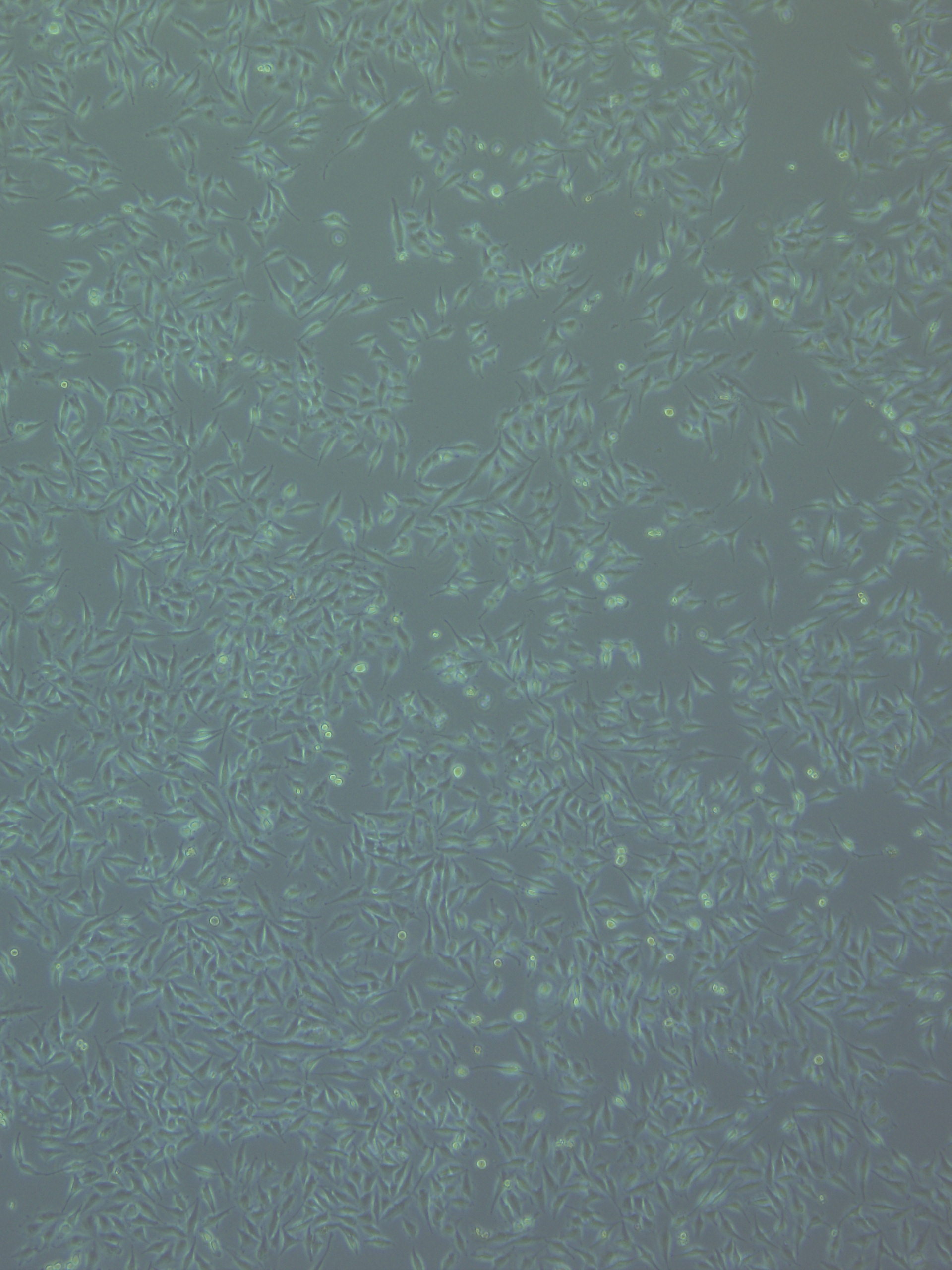

Supplement: Supplementary file 6 [file Image3.jpeg]

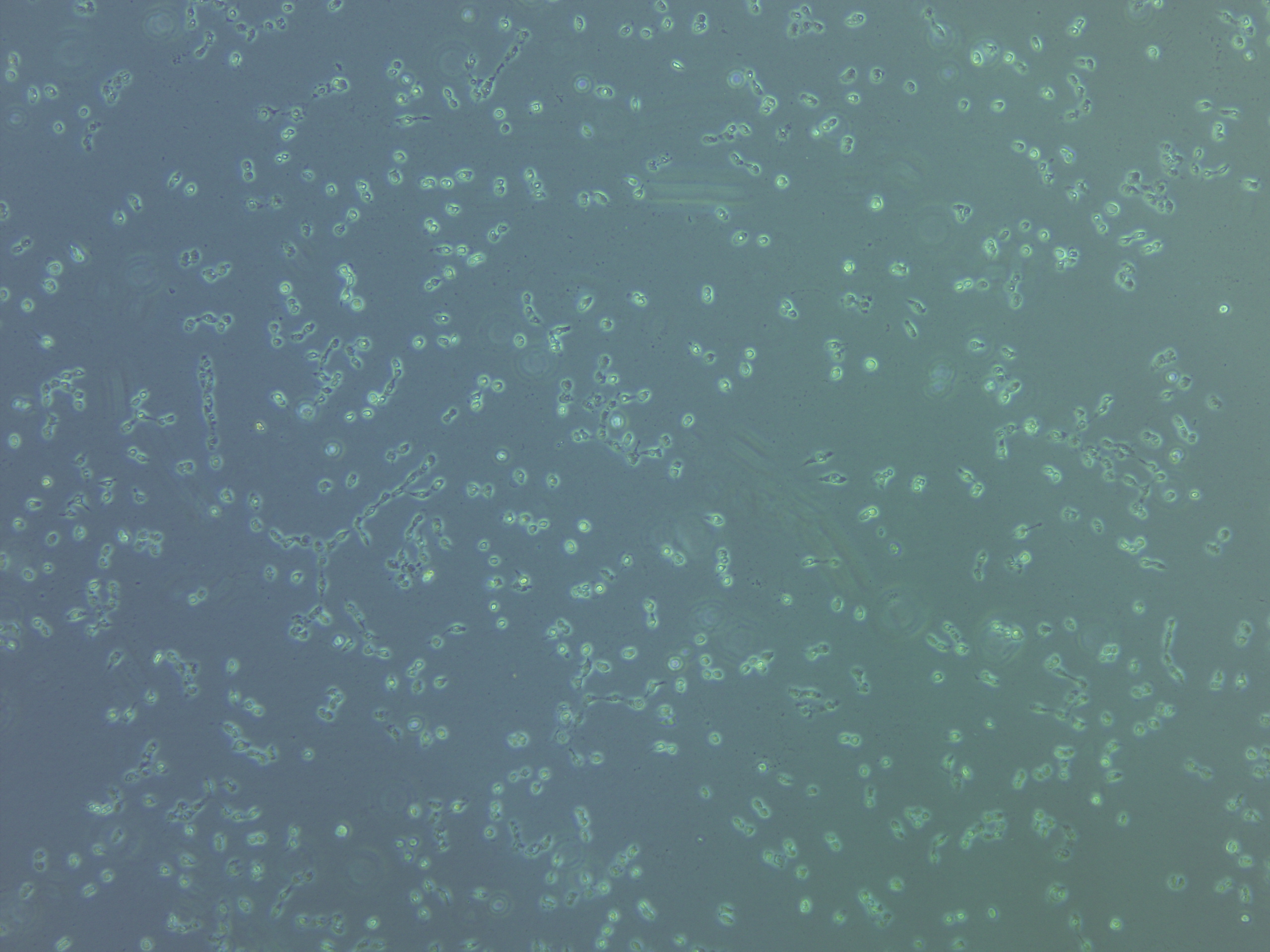

Supplement: Supplementary file 7 [file Image4.jpeg]

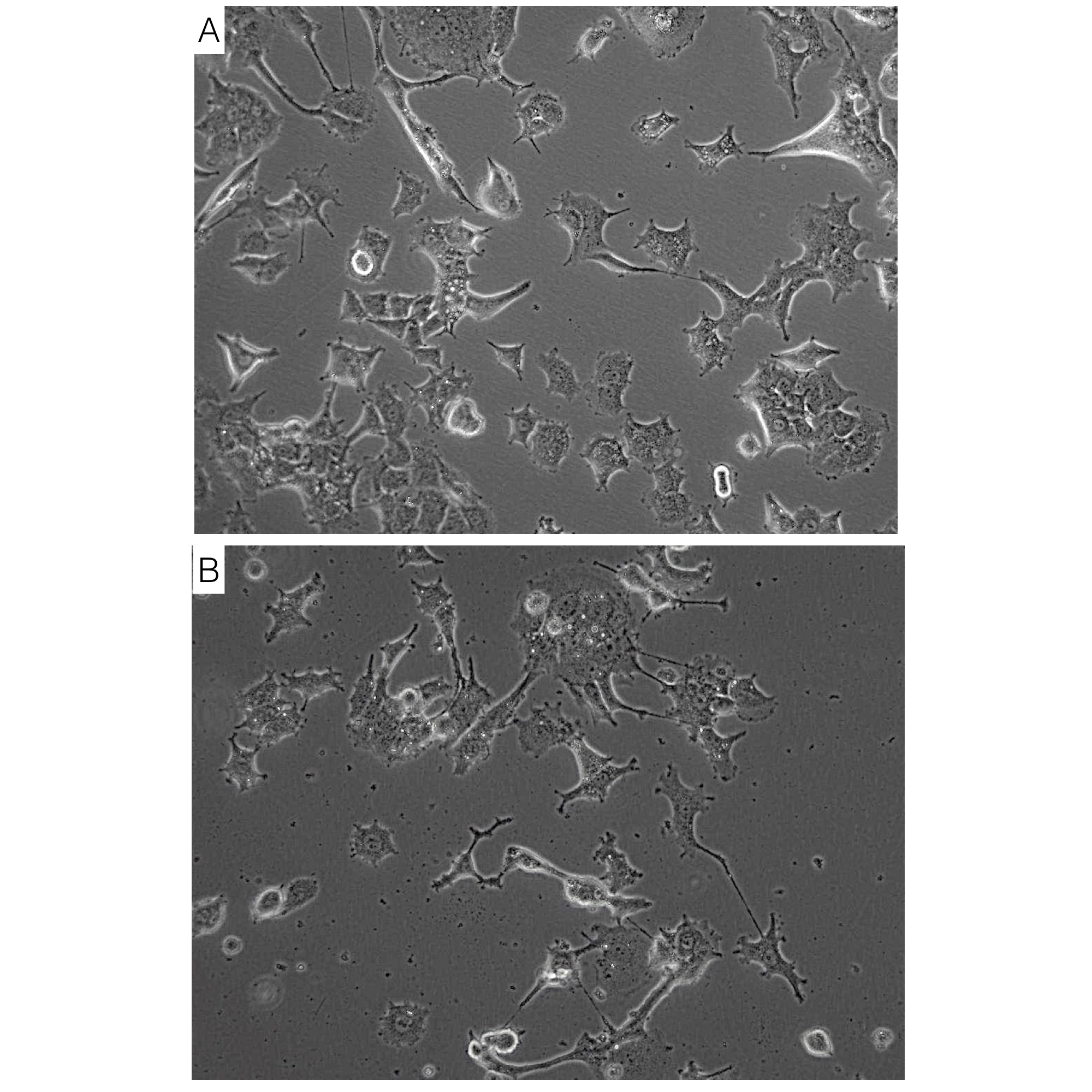

Supplement: Supplementary file 8 [file Image5.jpeg]

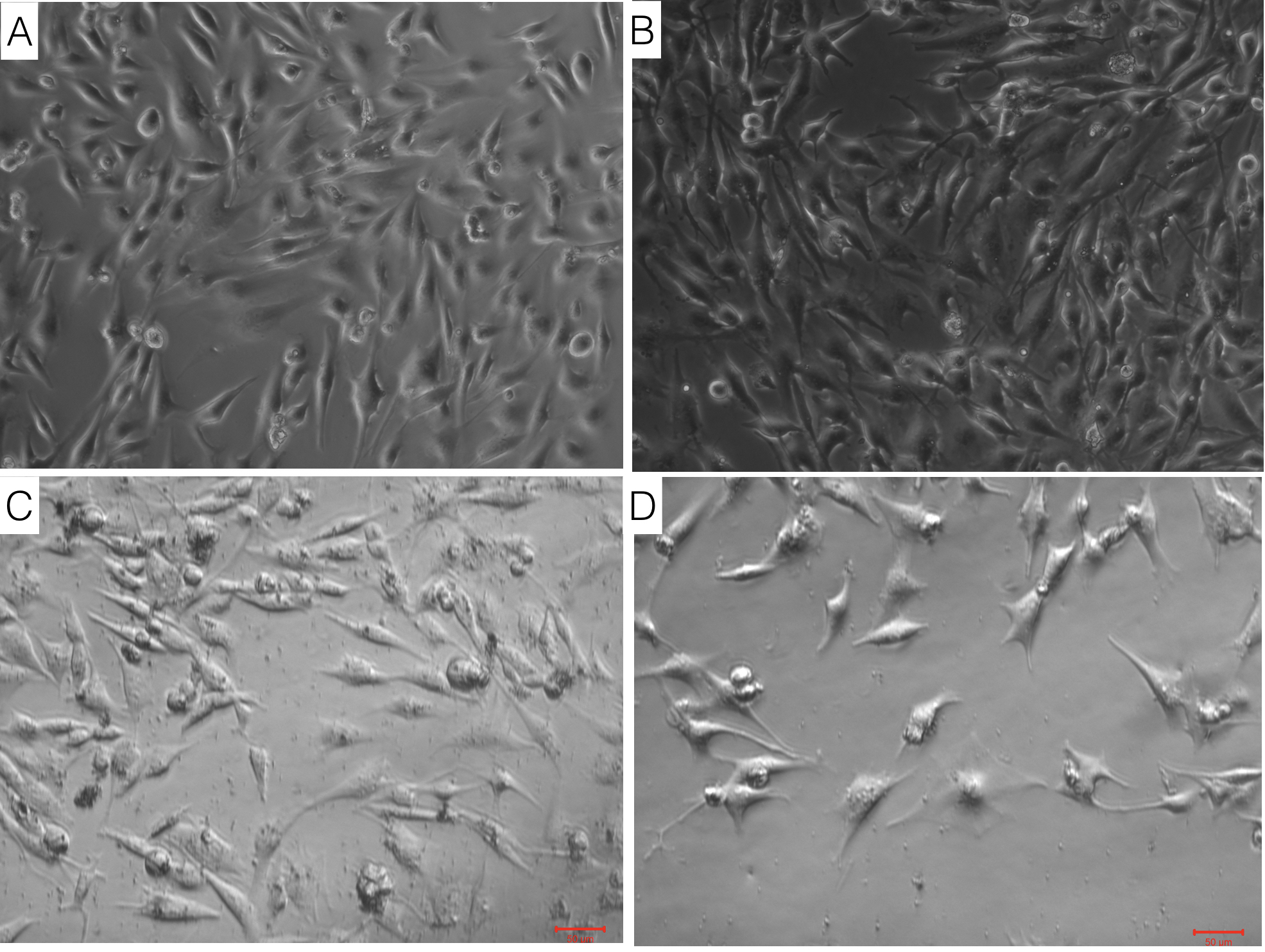

Supplement: Supplementary file 9 [file Image6.jpeg]

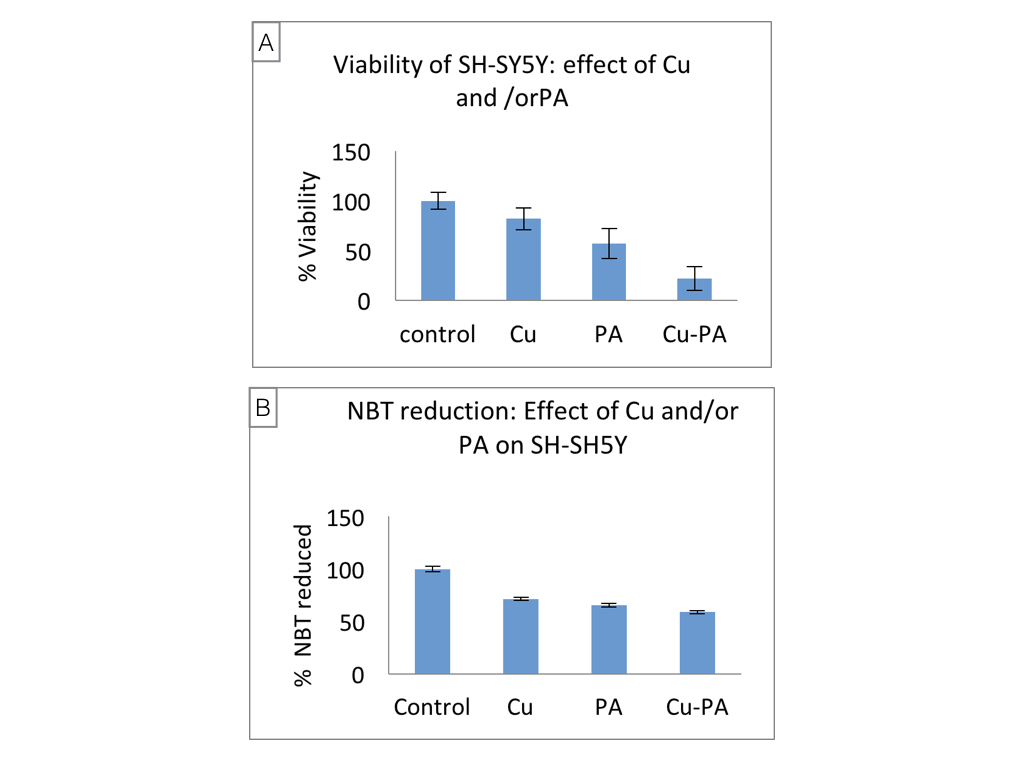

Supplement: Supplementary file 10 [file Image7.jpeg]
